# Lipid profile is associated with decreased fatigue in individuals with progressive multiple sclerosis following a diet-based intervention: Results from a pilot study

**DOI:** 10.1371/journal.pone.0218075

**Published:** 2019-06-18

**Authors:** Kelly Fellows Maxwell, Terry Wahls, Richard W. Browne, Linda Rubenstein, Babita Bisht, Catherine A. Chenard, Linda Snetselaar, Bianca Weinstock-Guttman, Murali Ramanathan

**Affiliations:** 1 Department of Pharmaceutical Sciences, State University of New York, Buffalo, New York, United States of America; 2 Department of Internal Medicine, University of Iowa, Iowa City, Iowa, United States of America; 3 Department of Neurology, University of Iowa, Iowa City, Iowa, United States of America; 4 Department of Biotechnical and Clinical Laboratory Sciences, State University of New York, Buffalo, New York, United States of America; 5 Department of Epidemiology, University of Iowa, Iowa City, Iowa, United States of America; 6 Department of Neurology, State University of New York, Buffalo, New York, United States of America; Foundation IRCCS Neurological Institute C. Besta, ITALY

## Abstract

**Purpose:**

To investigate associations between lipid profiles and fatigue in a cohort of progressive multiple sclerosis (MS) patients on a diet-based multimodal intervention.

**Methods:**

This pilot study included 18 progressive MS patients who participated in a prospective longitudinal study of fatigue following a diet-based multimodal intervention that included exercise, neuromuscular electrical stimulation and stress reduction. The diet recommended high intake of vegetables and fruits, encouraged consumption of animal and plant protein and excluded foods with gluten-containing grains, dairy and eggs. Fatigue was measured on the Fatigue Severity Scale (FSS) at baseline and every 3 months for 12 months. A lipid profile consisting of high-density lipoprotein cholesterol (HDL-C), low-density lipoprotein cholesterol (LDL-C), total cholesterol (TC) and triglycerides (TG) was obtained on fasting blood samples at baseline and 12 months.

**Results:**

FSS scores decreased from a baseline of 5.51 (95% CI: 4.86, 6.16) to a mean of 3.03 (95% CI: 2.23, 3.82) at 12 months (p < 0.001). At 12 months, increases in HDL-C (mean change: +6.0 mg/dl; 95% CI: 0.3, 12.0; p = 0.049) and decreases in BMI (mean change: -2.6 kg/m^2^; 95% CI: -3.6, -2.5; p < 0.001), LDL-C (mean change: -10.4 mg/dl; 95% CI:-19.7, -1.2; p = 0.029), TG (mean change: -29.2 mg/dl; 95% CI: -44.3, -14.2; p = 0.001), TG to HDL-C ratio (mean change: -0.6; 95% CI: -1.0, -0.3; p = 0.002) and TC to HDL-C ratio (mean change:-0.6; 95% CI: -1.0, -0.3; p = 0.003) were observed compared to baseline. Improvements in FSS were associated with increases in HDL-C (β = -0.05; 95% CI: -0.1, -0.0004; p = 0.048) and changes in TC (p = 0.005) from baseline to 12 months.

**Conclusions:**

Lipid profile variables are associated with improvements in fatigue in progressive MS patients on a diet-based multimodal intervention.

## Introduction

Fatigue is defined as “a subjective lack of physical and/or mental energy that is perceived by the individual or caregiver to interfere with usual or desired activities” [1]. Fatigue is a frequent and debilitating multiple sclerosis (MS) symptom [2]. Estimates of its prevalence range from 52% to 93% [3]. Fatigue affects MS patients’ quality of life independently of disability [4] and adversely affects their ability to work full-time.

The etiology of MS fatigue is considered multifactorial [5]. Fatigue can result from MS pathobiological processes, which cause blood-brain barrier breakdown, central nervous system inflammation, demyelination, lesion formation and neurodegeneration [5]. Fatigue can also include contributions from co-morbidities such as depression, physical and emotional stress and external factors, e.g., poor diet and lack of sleep [5]. However, MS fatigue appears to be a distinct clinical entity that differs from other causes of fatigue such as disability and depression [2].

Pharmacological options for treating MS-associated fatigue are limited. Patients are commonly prescribed modafinil or amantadine; fluoxetine is sometimes prescribed off-label. Anti-fatigue drugs have stimulant activity and are often associated with side effects. There is support for non-pharmacological options such as exercise, physical therapy with vestibular rehabilitation and cognitive behavioral therapy [6].

Some dietary interventions have shown promise for treating MS fatigue [7–10]. Yadav et al. showed that fatigue outcome measures were improved in relapsing-remitting MS patients on a very-low-fat, plant-based diet [7]. We previously reported that diet-based multimodal intervention supplemented with exercise, neuromuscular electrical stimulation (NMES) and stress reduction techniques was effective at reducing fatigue in progressive MS patients [8–10]. The study diet recommended high intake of vegetables and fruits, encouraged consumption of animal and plant protein and excluded foods with gluten-containing grains, dairy and eggs. However, the physiological mechanisms underlying the effectiveness of this multimodal intervention on fatigue are not known.

The associations between diet and lipid parameters have become delineated in recent meta-analyses conducted in the context of cardiovascular disease [11, 12]. The replacement of saturated fat by polyunsaturated and monounsaturated fats decreases TC, LDL-C and TG, whereas replacement of saturated fats by carbohydrates decreases TC and LDL-C, but increases TG. Saturated fat decreases and polyunsaturated fat increases the anti-inflammatory activity of HDL-C [13]. Replacing saturated fat with polyunsaturated or monounsaturated fat lowers HDL-C slightly. However, replacement with carbohydrates lowers HDL-C to a greater extent [12, 14].

Our working hypothesis that lipid and cholesterol pathways could be potential mediators of the effects of the study diet on fatigue was motivated by two factors. First, the study diet alters food macronutrient composition, which could affect metabolism, causing changes in lipid and cholesterol profiles. Second, an emerging body of evidence has demonstrated that metabolic changes [15, 16] underlie the immune and neurodegenerative pathophysiological processes of MS and that cholesterol biomarkers are associated with brain injury and disease progression in MS [17–21]. However, the roles, if any, of lipid and cholesterol pathways in MS fatigue have not been investigated.

The aims of this study were to characterize the changes in lipid and cholesterol biomarkers during the diet-based multimodal intervention and to investigate whether these biomarkers were associated with fatigue outcomes. We analyzed data obtained from a Phase 1 pilot trial of an integrative diet-based multimodal intervention (study diet, exercise, NMES, stress reduction) on fatigue in progressive MS patients. The aims of the trial were to assess the safety, patient adherence to the diet and other components of the study, effects and nutritional adequacy of the study diet for MS fatigue.

## Methods

### Study population and design

Data were obtained during a Phase 1 pilot trial of an integrative 12-month diet-based multimodal intervention (study diet, exercise, neuromuscular electrical stimulation, stress reduction) on fatigue in N = 20 progressive MS patients.

For this sub-study, participants with lipid profile data at baseline and 12 months were included for analysis (n = 18). Two participants were excluded, one who did not complete the study due to cognitive decline at six months and one lacking lipid profile data at 12 months.

### Inclusion and exclusion criteria

Details regarding the study diet, inclusion and exclusion criteria and protocols and procedures have been published [8–10]. Briefly, inclusion criteria were diagnosis of progressive MS confirmed by a neurologist specializing in MS; age 18–65 years; some level of gait impairment but ability to walk 25 feet with or without an assistive device; and an adult companion willing to assist with home exercise and neuromuscular electrical stimulation. Exclusion criteria were change in MS status in prior three months; abnormal renal or hepatic functions; current diagnosis of cancer (other than non-melanoma skin cancer); psychotic disorder; significant cognitive dysfunction; seizure disorder; heart block or abnormal rhythm; unstable heart disease; lung disease; diabetes requiring medication change in the past three months; implanted electronic device; antiplatelet or blood thinner medication; and vitamin D level > 150 ng/mL (or a vitamin D level >100 ng/mL combined with a blood calcium abnormal elevation > 10.2 mg/dL). Fatigue status was not part of the screening criteria.

### Study protocol and intervention

Ethical approval was obtained from the University of Iowa Human Subjects Institutional Review Board, which approved the study protocol on May 25, 2010. Written informed consent was obtained from all participants. The ClinicalTrials.gov Identifier is NCT01381354.

Participants were first enrolled into a two-week run-in period during which they were educated about the study diet. A stretching exercise program was designed for each participant. Participants were asked to follow the study diet and perform stretches during the run-in period and complete daily food and exercise logs. During the second visit a test electrical stimulation session was conducted.

Participants who adhered to the study diet for seven consecutive days during the run-in period and could tolerate electrical stimulation were enrolled into the 12-month study.

### Study diet

The study diet was the salient component of the multimodal intervention. The diet consisted of recommended, excluded and encouraged foods. Recommended foods were three daily servings (cup-equivalents) each of green leafy vegetables, sulfur-rich vegetables and deeply colored fruits and vegetables. Excluded foods were gluten-containing grains, dairy and eggs. Encouraged foods included daily servings of animal protein (4 ounces or more), plant protein (4 ounces or more), omega-3 oils (2 tablespoons), choice of non-dairy milks such as soy, almond, rice and coconut, nutritional yeast (1 tablespoon), kelp (1/4 teaspoon powder or two capsules), spirulina/chlorella/Klamath blue–green algae (¼ to 1 teaspoon or four to eight capsules). Only two servings of gluten-free grains or starchy foods were allowed each week. Participants were advised to eat until satiety. Weight loss was not a goal of the study. If participants lost more than 10% of their body weight, their primary care physician was notified and the study team worked with participants to increase consumption of more high Calorie foods. No participant engaged in fasting.

Dietary supplements thought to be beneficial for MS and fatigue were recommended to participants [8] including up to four grams of fish oil per day. The other supplements recommended included B vitamins (thiamine, riboflavin, niacinamide, methylcobalamin, methyl folate), mitochondrial performance supplements (coenzyme Q10, *α*-lipoic acid sustained release, taurine, creatine monohydrate, L-acetyl carnitine), vitamins, minerals and amino acid dietary supplement (Pinnaclife Full Spectrum, Pinnaclife, Coralville, IA), amino acid dietary supplement (Pinnaclife Essential), vitamin and mineral supplement (magnesium, calcium plus vitamin D3, Pinnaclife Mineral Boost) and soluble fiber supplement (Pinnaclife Cleanse). Details of the composition of the Pinnaclife Full Spectrum, Pinnaclife Essential, Pinnaclife Mineral Boost and Pinnaclife Cleanse supplements are provided in S1 Supplementary Data. Participants were allowed to refuse or discontinue taking supplements at any time and for any reason [8].

The eating plan was first published in a case report of a patient with secondary progressive MS who experienced marked reduction in fatigue severity and disability [22], which then led to subsequent safety and feasibility studies [8]. In our prior publications, the study diet was referred to as a modified Paleolithic diet; however, it is simply referred to as the study diet in this report.

### Exercise, neuromuscular electrical stimulation and stress reduction

A home-based exercise program that included stretches and strengthening exercises for leg and trunk muscles was designed for each subject. Most strengthening exercises were performed along with neuromuscular electrical stimulation (exercise-NMES program) to facilitate muscle contraction and movement. Initially, participants were asked to perform 10–20 repetitions of a muscle group exercise within 10 minutes of electrical stimulation. The daily repetitions of stretches and strengthening exercises, and duration of electrical stimulation were progressively increased as participants’ tolerance improved. Participants were asked to perform stretches and exercise-NMES at least five days per week. Details of types of exercises and electrical stimulation have been described previously [8, 9].

For stress reduction, participants were instructed to meditate and self-massage hands, feet, and face for a recommended duration of 20 minutes daily [8, 9].

### Clinical and laboratory assessments

Fatigue was measured on the Fatigue Severity Scale (FSS) at baseline and 3, 6, 9 and 12 months. Disability was assessed using the Kurtzke Expanded Disability Status Scale (EDSS) by the study neurologist at baseline and 12 months.

Body mass index (BMI) values were calculated from corresponding weight and height measurements as the ratio of weight in kg to the square of height in meters.

A complete lipid profile consisting of TG, TC and HDL-C was obtained from a clinical laboratory on fasting blood samples obtained at baseline and 12-month visits. LDL-C was calculated using the Friedewald equation [23]. Ratios for TC-to-HDL-C and TG-to-HDL-C were computed from the laboratory values.

### Data analyses

Average adherence, average number of servings per day of recommended foods and average number of servings per day of excluded foods were calculated from participants’ daily logs [9]. Participants were considered adherent to the study diet on a study day if they consumed any recommended foods and did not consume an excluded food [9]. Participants were considered adherent to exercise-NMES for each day that they performed either exercise or NMES, or both [9]. The average duration in minutes of total daily exercise, NMES, meditation and massage were calculated from food and exercise logs every three months. We refer to diet adherence, servings per day of recommended foods, servings per day of excluded foods, exercise-NMES adherence, duration in minutes per day of exercise, NMES, massage, or meditation collectively as intervention-adherence variables.

Calorie and macronutrient data were derived from the 2007 semi-quantitative Harvard Food Frequency Questionnaire [24], which was completed by each participant at baseline and 12 months. The questionnaire collected information about food and supplement intake during the past year. Questionnaires were analyzed by Harvard between October 2011 and April 2014 using the Harvard nutrient databases.

Statistical analyses were conducted with the SPSS statistical program (version 24). Results are expressed as number and percent (n (%)) or means and standard deviations (SD) or means and 95% confidence intervals (95% CI). All baseline, 12-month and change score variables were assessed for normality and suitability for parametric analyses using graphical displays. Statistical significance was set at p < 0.05.

When data was gathered at all visits, the clinical outcome measure of interest was the change in FSS scores over time, which was comprised of FSS scores obtained at baseline and 3, 6, 9 and 12 months. Linear mixed effects analysis with a random intercept for participant were used to investigate the associations of the change in FSS scores with each of the intervention-adherence variables over time. The FSS scores (dependent variable) and the intervention-adherence variables (independent variables) obtained at the baseline, 3, 6, 9 and 12-month visits were the repeated variables. Age, gender, baseline EDSS, and time were fixed-effect independent variables.

Because lipid profile data was only collected at baseline and month 12, the clinical outcome measure of interest was the mean change in FSS (FSS score at month 12 minus the FSS score at baseline). All clinical, adherence and nutrient intake variables and their associations with lipid profile were assessed as mean differences (month 12 value−baseline value).

A paired *t*-test was used to assess changes in participants’ nutrient intake and lipid profile variables (HDL-C, LDL-C, TC, TG, TG-to-HDL-C ratio, TC-to-HDL-C ratio and BMI) between baseline and 12 months. Results were confirmed using a t-test for change scores. The p-values for nutrient intake variables were adjusted for multiple testing with the Benjamini-Hochberg method in the R statistical program.

The associations of mean nutrient changes and mean lipid profile changes, using only baseline and 12-month values, were assessed using general linear model methods with the lipid variable as the outcome and the nutrient intake as the independent variable. The results are displayed as β coefficients, 95% confidence intervals, and an associated *p*-value. The magnitude of the β coefficient (positive or negative) represents the degree of change in the outcome variable for every 1-unit of change in the predictor variable. The linear models were generated with and without the baseline lipid value as an adjustment variable

The association of change in FSS scores (FSS baseline−FSS at 12 months) with changes in lipid profile variables (HDL-C, LDL-C, TC, TG and BMI) from baseline to 12 months used general linear model methods as described for nutrient and lipid profile analysis. The FSS change from baseline to 12 months was the dependent variable and the change in lipid profile variable of interest from baseline to 12 months was the independent variable. In additional analyses, we investigated the impact of adjusting for the average intervention-adherence variables over 12 months on these associations. In these analyses, FSS change from baseline to 12 months was the dependent variable and the change in the lipid profile variable of interest from baseline to 12 months, and the average intervention-adherence variable of interest over 12 months were treated as independent variables.

## Results

### Demographic and clinical characteristics

**Fig 1:**
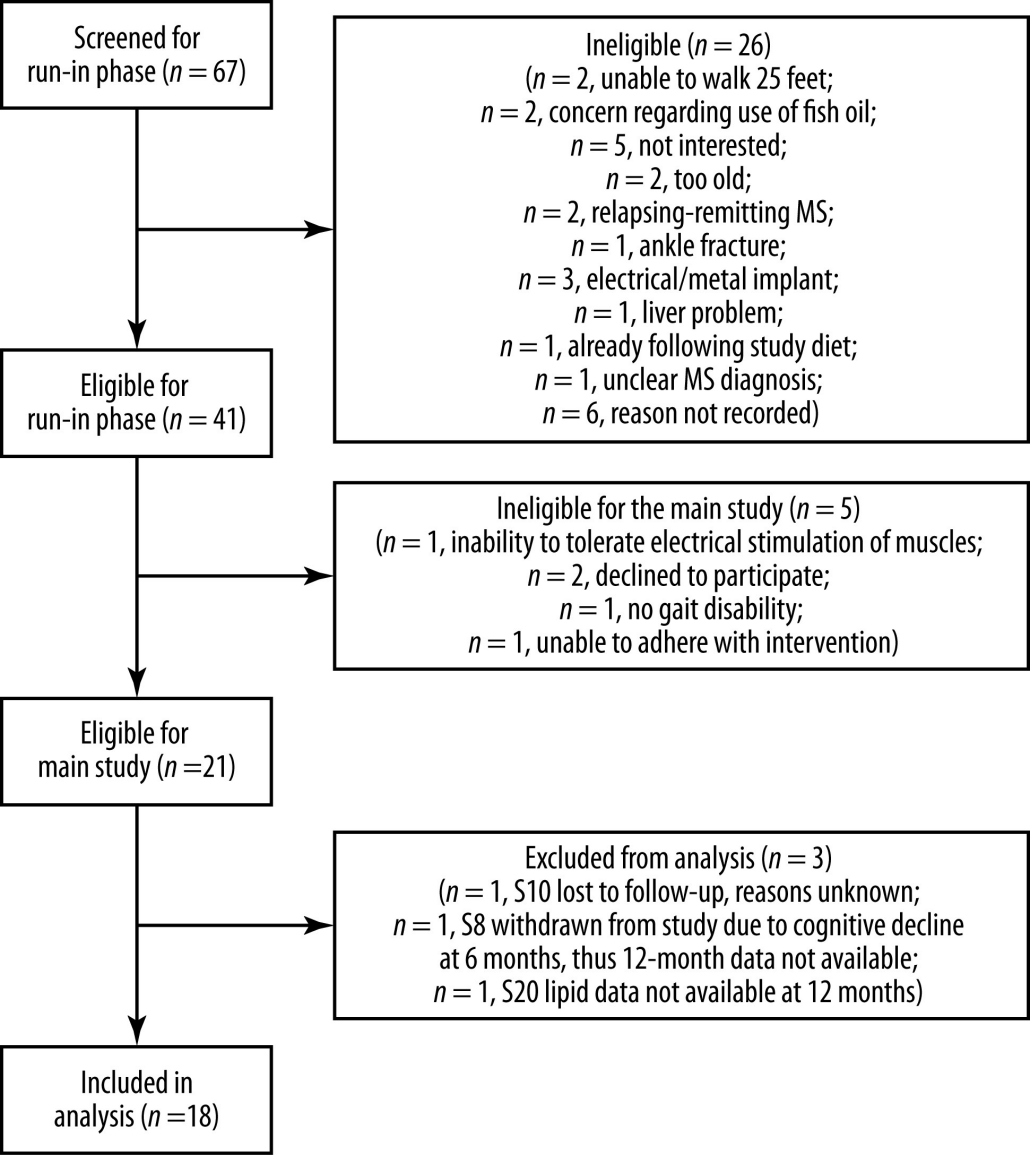
CONSORT diagram for the lipid profile and fatigue sub-study.

In this sub-study, 18 progressive MS patients (16 secondary progressive MS and two primary progressive MS patients) were included. Fig 1 shows the CONSORT diagram. Baseline clinical and demographic characteristics of participants are shown in Table 1. As this was a cohort of progressive MS patients, the majority (n = 13, 72.2%) used a walking aid. Sixteen of the 18 study participants (89%) had a baseline FSS score ≥ 4.0. Twelve of the 18 subjects were not on any disease-modifying treatment (DMT) and six patients were on approved DMT. Treatment decisions were at the discretion of the patients’ treating neurologists. No changes to the patients’ disease-modifying treatments (DMT) were made as part of the study. The lipid-lowering medications reported were: simvastatin (1 patient) and red yeast rice supplement (1 patient).

**Table 1:** Baseline demographic and clinical characteristics of study participants.

Characteristic	*n*	Mean (SD) Or *n* (%)
**Age, years**	18	51.8 (6.7)
**Gender, % Female**	18	13 (72.2)
**Body mass index, kg/m**^**2**^	18	24.5 (3.2)
**Race:**	18	
Caucasian		17 (94.4)
Hispanic		1 (5.6)
**Education:**	18	
High school		1 (5.6)
Some college		6 (33.3)
4-year degree		4 (22.2)
> College		7 (38.9)
**Diagnosis:**	18	
SP-MS		16 (88.9)
PP-MS		2 (11.1)
**Walking aid**	18	13 (72.2)
**Disease duration, years**	18	13.2 (7.6)
EDSS*	18	6.2 (1.0)
**Fatigue Severity Scale**	18	5.5 (1.3)
**Disease modifying treatments:**	18	
No treatment		12 (67%)
Interferon-beta		4 (22%)
Glatiramer acetate		1 (6%)
Natalizumab		1 (6%)

* One patient had EDSS 8.0. This EDSS assessment could be discrepant because the patient was exhausted during the neurological examination, which was conducted on the same day after the 25-foot walk. This patient completed the get-up-and-go test (time taken: 172 seconds) and 25-foot walk (speed: 3.7 cm/s) test at baseline, though with great difficulty.

**Abbreviations**: SP-MS: Secondary progressive multiple sclerosis; PP-MS: Primary progressive multiple sclerosis; EDSS: Expanded Disability Status Scale (possible range 0–10); Fatigue Severity Scale (FSS9; possible range 1–7)

### Calorie and macronutrient intake

Daily calorie and macronutrient intake at baseline and 12 months and corresponding changes are summarized in Table 2.

**Table 2:** Average daily calorie intake and macronutrient distributions at baseline and 12 months.

Variable	Baseline Means (95% CI) ^A^	12 Months Means (95% CI) ^A^	Mean Changes (95% CI) ^B^	p-value ^C^	q-values^k^
Energy, kcal	1767 (1514, 2021)	1288 (1105, 1472)	-479 (-780, -178)	0.004	0.007
Percent calories from					
Protein, %	15.1 (13.6, 16.6)	20.3 (17.7, 22.9)	5.2 (2.7, 7.7)	0.0004	0.001
Carbohydrate, %	48.4 (45.3, 51.6)	37.3 (33.5, 41.1)	-11.1 (-15.3, -7.0)	< 0.001	< 0.002
Total sugar, %	22.0 (18.5, 25.4)	19.4 (16.8, 22)	-2.5 (-7.1, 2.0)	0.26	0.30
Added sugar, %	11.9 (9.1, 14.6)	5.2 (3.2, 7.2)	-6.7 (-10.3, -3.1)	0.001	0.002
Natural sugar, %	10.1 (7.7, 12.4)	14.2 (12.2, 16.2)	4.1 (1.1, 7.2)	0.01	0.02
Fat, %	34.9 (31.6, 38.2)	43.8 (39.6, 48.0)	9.0 (4.0, 13.9)	0.001	0.002
Saturated fat, %	11.4 (10, 12.9)	8.6 (7.8, 9.4)	-2.8 (-4.4, -1.1)	0.002	0.0046
Trans fat, %	1.2 (1.0, 1.3)	0.6 (0.5, 0.7)	-0.6 (-0.8, -0.4)	< 0.0001	< 0.0005
Monounsaturated fat, %	12.7 (11.4, 14.0)	18.9 (16.6, 21.1)	6.2 (3.5, 8.8)	< 0.0002	< 0.0008
Polyunsaturated fat, %	7.5 (6.2, 8.8)	12.5 (10.1, 14.9)	5.0 (2.4, 7.7)	< 0.001	0.002
Alcohol, %	3.7 (0.6, 6.8)	2.5 (0.4, 4.5)	-1.2 (-3.3, -0.9)	0.23	0.29
Cholesterol, mg/1000 kcal	108 (93, 122)	114 (91, 137)	7.0 (-13.0, 26.0)	0.48	0.50
Dietary fiber, g/1000 kcal	11.1 (9.5, 12.6)	18.6 (17.0, 20.2)	7.5 (5.8, 9.3)	< 0.0001	< 0.0005
Glycemic index ^J^	51.9 (49.6, 54.1)	41.5 (38.3, 44.8)	-10.4 (-13.4, -7.3)	< 0.0001	< 0.0005
Glycemic load ^J^	110 (94, 126)	51.6 (39.1, 64.0)	-58.1 (-75.8, -40.4)	< 0.0001	< 0.0005
Omega-3 fatty acids, fish ^D^ g/1000 kcal	0.08 (0.05, 0.11)	0.3 (0.1, 0.5)	0.2 (0.1, 0.3)	< 0.005	< 0.008
Omega-3 fatty acids, fish and fish oil, ^E^ g/1000 kcal	0.2 (0.1, 0.3)	0.7 (0.5, 0.9)	0.5 (0.3, 0.8)	< 0.0002	< 0.0008
Omega-3 fatty acids total, ^F^ g/1000 kcal	1.0 (0.7, 1.3)	2.7 (2.1, 3.3)	1.7 (1.2, 2.1)	< 0.0001	< 0.0005
Omega-3 fatty acids total without supplements, ^G^ g/1000 kcal	0.8 (0.6, 0.9)	1.8 (1.4, 2.3)	1.0 (0.6, 1.4)	0.0001	0.0005
Omega-6 fatty acids, ^H^ g/1000 kcal	7.0 (5.9, 8.0)	11.7 (9.2, 14.1)	4.7 (1.8, 7.5)	<0.003	< 0.006
Omega-6 fatty acids without supplements, ^I^ g/1000 kcal	6.9 (5.9, 8.0)	11.5 (9.0, 14.0)	4.6 (1.7, 7.4)	<0.004	< 0.007
Protein, g	68.0 (54.7, 81.2)	66.5 (52.4, 80.7)	-1.4 (-19.7, 16.8)	0.87	0.87
Carbohydrate, g	212.7 (180.6, 244.8)	119.6 (98.4, 140.9)	-93.1 (-129.4, -56.7)	<0.0001	< 0.0005
Total sugar g	99.4 (74.1, 124.7)	61.4 (50.8, 71.9)	-38.0 (-9.3, -66.7)	0.012	0.018
Added sugar g	52.8 (37.4, 68.2)	16.4 (9.8, 22.9)	-36.4 (-54.-18.9)	0.004	0.007
Natural sugar g	46.6 (29.9, 63.3)	45.0 (36.6, 53.4)	-1.6 (-21.0, 17.8)	0.86	0.87
Total fat g	69.0 (56, 82.1)	63.0 (52.1, 73.8)	-6.1 (-22.1, 10.0)	0.44	0.47
Saturated fat g	23.2 (17.4, 29.1)	12.6 (10.1, 15.1)	-10.6 (-17.1.-4.1)	0.003	0.006
Trans fat g	2.4 (1.9, 2.8)	0.9 (0.6, 1.1)	-1.5 (-2.0,-1.0)	<0.0001	< 0.0005
Monounsaturated fat g	24.9 (20.7, 29.2)	27.3 (22.2, 32.4)	2.3 (-3.8, 8.5)	0.43	0.47
Polyunsaturated fat g	14.5 (11.3, 17.6)	17.6 (13.8, 21.3)	3.1 (-1.9, 8.1)	0.20	0.26
Alcohol g	8.3 (2.4, 14.2)	3.6 (1.0, 6.3)	-4.7 (-8.5, -0.9)	0.018	0.025
Cholesterol mg	196 (151.3, 240.3)	151 (110, 192)	-45 (-100, 10)	0.10	0.13
Dietary fiber g	18.9 (16.0, 21.7)	23.4 (20.5, 26.4)	4.5 (1.3, 7.8)	0.009	0.014
Omega-3 fatty acids fish ^D^ g	0.15 (0.1, 0.2)	0.4 (0.2,0.5)	0.2 (0.1, 0.3)	0.005	0.008
Omega-3 fatty acids fish and fish oil ^E^ g	0.3 (0.2, 0.4)	0.8 (0.6, 1.1)	0.5 (0.3, 0.8)	0.001	0.002
Omega-3 fatty acids total ^F^ g	1.7 (1.3, 2.2)	3.2 (2.6, 3.7)	1.4 (0.8, 2.1)	0.0002	0.0008
Omega-3 fatty acids total without supplements ^G^ g	1.4 (1.1, 1.7)	2.2 (1.7, 2.8)	0.8 (0.2, 1.5)	0.016	0.023
Omega-6 fatty acids ^H^ g	12.2 (9.5, 14.9)	14.9 (11.3, 18.6)	2.7 (-2.1, 7.6)	0.25	0.30
Omega-6 fatty acids without supplements ^I^ g	12.1 (9.4, 14.9)	14.8 (11.1, 18.4)	2.6 (-2.3, 7.5)	0.27	0.31

^A^ Unadjusted means and 95% confidence intervals(CI), except where noted as mean percentages and 95% CI

^B^ The means for baseline and 12 month changes is calculated by taking the differences for each participant and then calculating the mean of the individual changes

^C^ p-values are for the mean of individual differences and generated via *t*-tests

^D^ Sum of 20:5 (n-3) eicosapentaenoic acid and 22:6 (n-3) docosahexaenoic acid from food (fish) only

^E^ Sum of 20:5 (n-3) eicosapentaenoic acid and 22:6 (n-3) docosahexaenoic acid from food and supplements

^F^ Sum of 18:3(n-3) alpha-linolenic acid, 20:3(n-3) eicosatrienoic acid, 20:5(n-3) eicosapentaenoic acid, 22:3(n-3) 13c,16c,19c-docasatrienoic fatty acid, 22:5(n-3) docosapentaenoic acid, and 22:6(n-3) docosahexaenoic acid from food and supplements

^G^ Sum of 18:3(n-3) alpha-linolenic acid, 20:3(n-3) eicosatrienoic acid, 20:5(n-3) eicosapentaenoic acid, 22:3(n-3) 13c,16c,19c-docasatrienoic fatty acid, 22:5(n-3) docosapentaenoic acid, and 22:6(n-3) docosahexaenoic acid from food only

^H^ Sum of 18:2(n-6) linoleic acid, 18:3(n-6) gamma-linolenic acid, 20:2(n-6) eicosadienoic acid, 20:3(n-6) dihomo-gamma-linolenic acid, 20:4(n-6) arachidonic acid, 22:2(n-6) docosadienoic acid, and 22:4(n-6) adrenic acid from food and supplements

^I^ Sum of 18:2(n-6) linoleic acid, 18:3(n-6) gamma-linolenic acid, 20:2(n-6) eicosadienoic acid, 20:3(n-6) dihomo-gamma-linolenic acid, 20:4(n-6) arachidonic acid, 22:2(n-6) docosadienoic acid, and 22:4(n-6) adrenic acid from food only

^J^ glucose reference.

^k^ q-values after Benjamini-Hochberg adjustment.

Decreases were observed for mean energy. To control for energy differences, nutrient values expressed as percent energy or per 1000 kcals were compared. Increases were seen for percent energy from total fat, monounsaturated and polyunsaturated fats, protein and natural sugar. Decreases were observed for percent Calories from carbohydrate, added sugar, saturated and trans fat.

The reduction in energy intake at 12 months is primarily due to the decreased carbohydrate intake (93 grams) from the elimination of grains and dairy, plus smaller reductions in saturated and trans fat and alcohol intake. Mean added sugar intake at 12 months was reduced to 5.2% of energy with 15 of 18 (83%) participants consuming less than the 10% maximum recommended by the Dietary Guidelines for Americans [25]. Glycemic index and glycemic load also decreased.

The percent energy from saturated fat at 12 months was significantly reduced from baseline with 15 of 18 (83%) participants consuming less than the 10% recommended by the Dietary Guidelines for Americans [25]. The reductions in saturated and trans fats were not compensated for with a significant increase in grams of monounsaturated or polyunsaturated fat; however, when monounsaturated and polyunsaturated fat were expressed as percent energy, significant increases were observed for both.

A significant increase in protein as a percent of energy was observed, although the protein intake in grams was unchanged.

Mean cholesterol intakes at baseline and 12 months were unchanged with eight of 18 (44%) and seven of 18 (39%) participants, respectively, consuming more than the 200 mg recommended by the National Lipid Association for reducing dyslipidemia [26]. Dietary fiber, omega-3 fatty acids per gram and grams/1000 kcal, and omega-6 fatty acids grams/1000 kcal increased.

The observed differences in nutrient intake are consistent with changes expected from the study diet guidelines to reduce or eliminate grains (carbohydrate) and dairy (carbohydrate and saturated fat), while encouraging consumption of fiber-containing vegetables and fruits plus foods and supplements containing omega-3 fatty acids.

### Changes in FSS over 12 months

**Fig 2:**
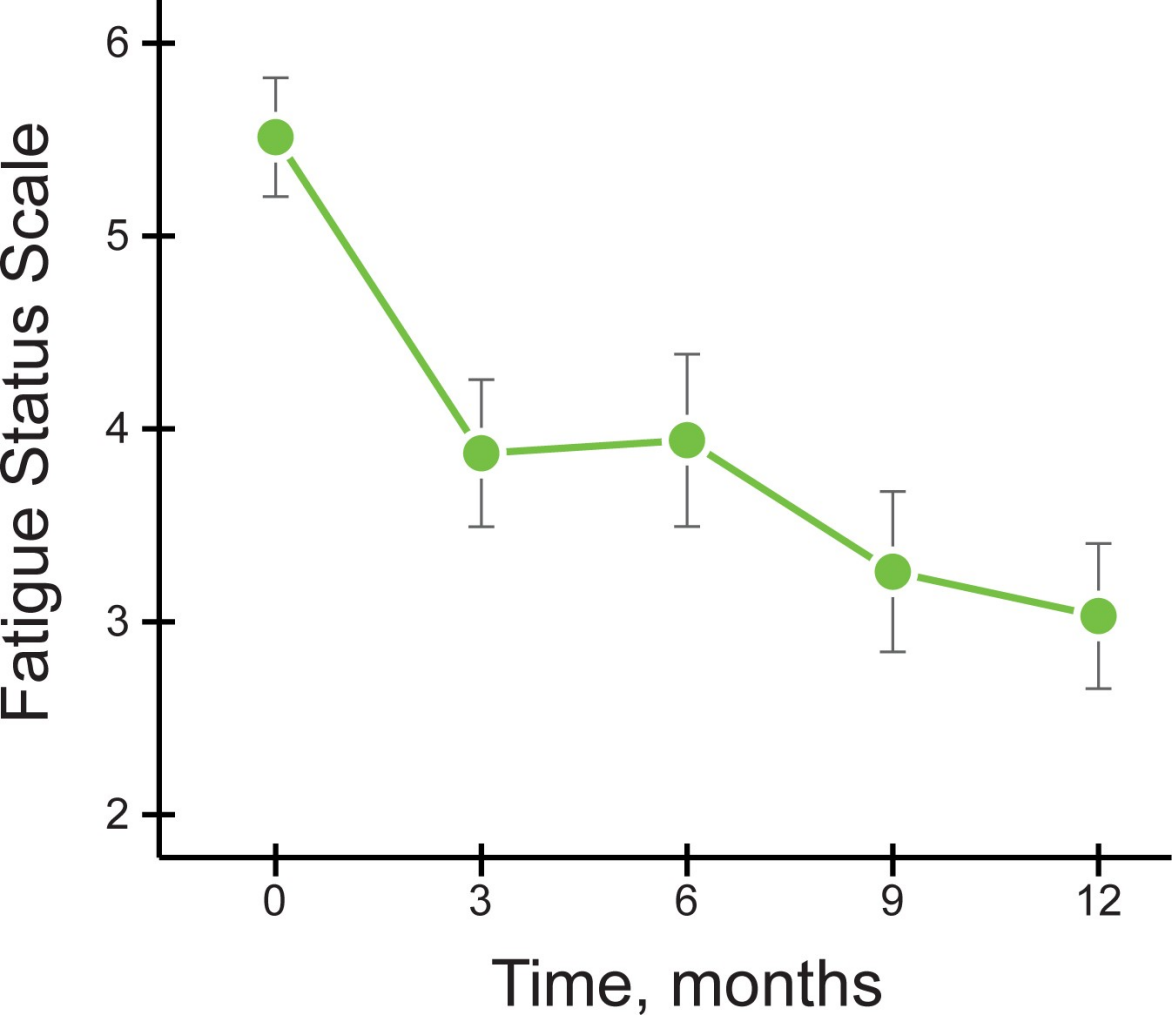
Mean Fatigue Status Scale (FSS) scores at baseline (0 months), 3, 6, 9 and 12 months. Error bars are standard error of the mean.

The mean decrease in FSS score was 2.48 (95% CI: -3.24, -1.73), from a baseline of 5.51 (95% CI: 4.86, 6.16; SD: 1.3) to a mean of 3.03 (95% CI: 2.23, 3.82; SD: 1.6) at 12 months (p < 0.001). In repeated measures analyses, the FSS at 3, 6, 9 and 12 months (all p < 0.001) were decreased compared to baseline FSS (Fig 2).

Baseline FSS in the DMT group (mean FSS: 5.7, 95% CI: (4.6, 6.9)) was similar (p = 0.62) to that in the group not on DMT (mean FSS: 5.4, 95% CI: (4.6, 6.2)). There were no differences (p = 0.88) in the FSS change between the DMT group (mean FSS change: -2.6, 95% CI: (-3.8, -1.3)) and the group not on DMT (mean FSS change: -2.4, 95% CI: (-3.4, -1.4)).

We next assessed whether the diet component of our intervention was a salient contributing factor to the improvements in fatigue over time. The decrease in FSS scores over time were associated with the greater mean servings of recommended foods (β = -0.28, 95% CI: (-0.35, -0.20), p < 0.001) and fewer mean servings of excluded foods (β = 0.31, 95% CI: (0.21, 0.42), p < 0.001) in the diet.

We then investigated the impact of the exercise, NMES, massage and meditation components on changes in fatigue over time. Fatigue change over time was not associated with average exercise-NMES adherence (p = 0.29), average daily minutes of exercise (p = 0.064), NMES (p = 0.29), average daily minutes of massage (p = 0.50) or average daily minutes of meditation (p = 0.96).

### Lipid profile changes

**Fig 3:**
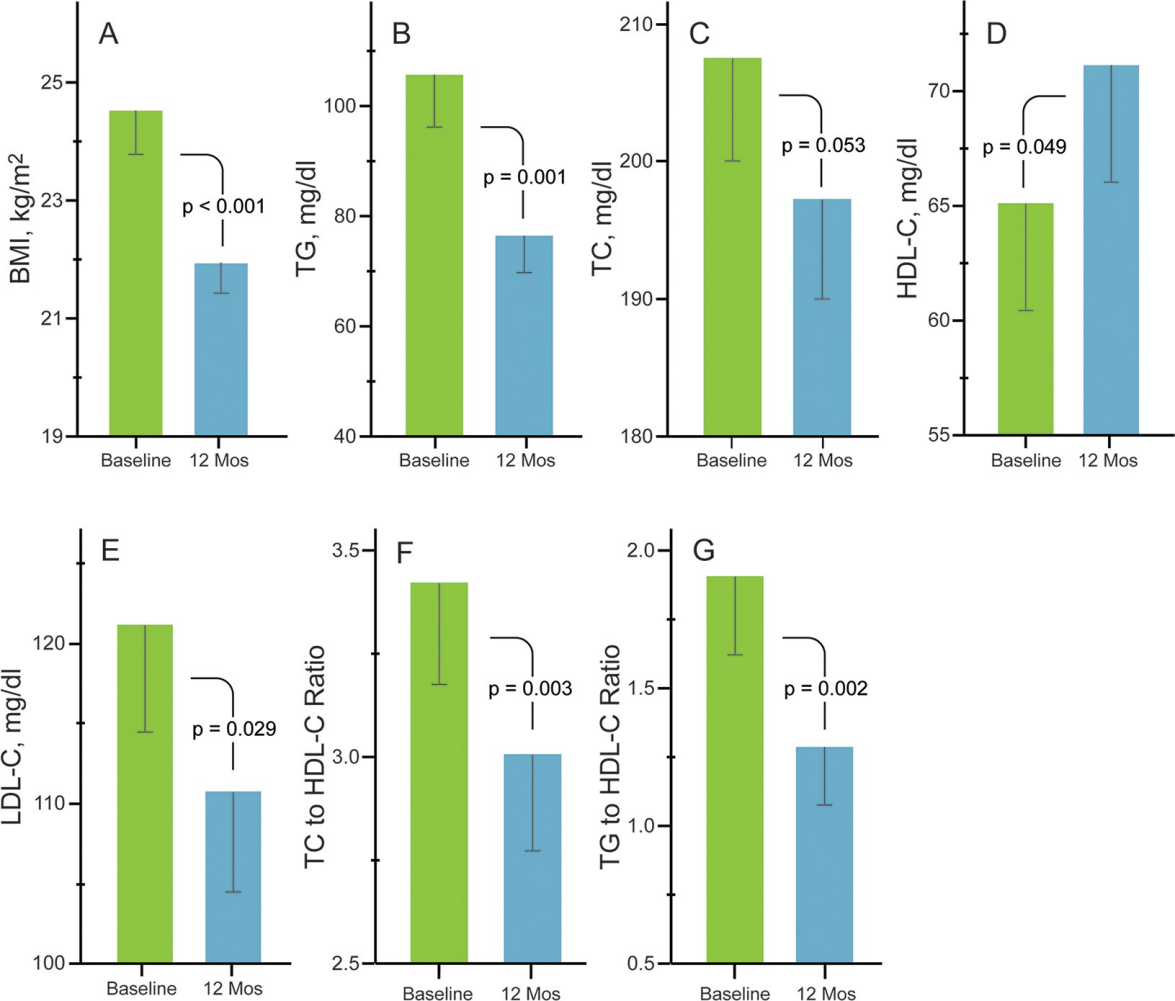
Body mass index (A), triglycerides (B), total cholesterol (C), high density lipoprotein cholesterol (D), low density lipoprotein cholesterol (E), total cholesterol to high density lipoprotein cholesterol ratio (F) and triglycerides to high density lipoprotein cholesterol ratio (G) at baseline and 12 months. Error bars are standard error of the mean. P-values generated via paired *t*-tests.

Improvements in BMI (Fig 3A) and all lipid profile variables (Fig 3B–3E) were observed over 12 months. Mean BMI decreased (mean change: -2.6 kg/m^2^, 95% CI: (-3.6, -2.5)) and a significant decrease was seen in triglyceride levels. HDL-C levels increased and LDL-C levels and TC levels decreased. There were significant decreases in TC-to-HDL-C ratio and in TG-to-HDL-C ratio. The mean changes (95% CI) are summarized in Table 3 and *p*-values are shown in Fig 3.

**Table 3:** Associations ^A^ of nutrient intake changes with lipid profile changes.

		Unadjusted β Coefficients (95% CI) and *p*-values (except where noted)					
		TC	HDL-C	LDL-C	TG	TC to HDL-C ratio	TG to HDL-C ratio
Lipid Profile Mean Changes (95% CI), mg/dl ^B^ → Intake Mean Changes (95% CI) ^C^ ↓		-10.2 (-20.7, 0.2)	+6.0 (0.3, 12.0)	-10.4 (-19.7, -1.2)	-29.2 (-44.3, -14.2)	-0.4 (-0.6, -0.1)	-0.6 (-1.0, -0.3)
Cholesterol, mg	-45 (-100, 10)						
Total fat, g	-6.1 (-22.1, 10.0)	0.13 (0.04, 0.21) 0.005			0.48 (0.03, 0.9) 0.036		
Saturated fat, g	-10.6 (-17.1.-4.1)						
Monounsaturated fat, g	2.3 (-3.8, 8.5)						
Polyunsaturated fat, g	3.1 (-1.9, 8.1)					-0.03 (-0.1, 0.0) 0.050	
Trans fat, g	-1.5 (-2.0,-1.0)				15.6 (0.7, 30.6) 0.042		
Omega-3 fatty acids, fish and fish oil, g ^D^	0.5 (0.3, 0.8)						
Omega-3 fatty acids total, g ^E^	1.4 (1.1, 1.7)						
Omega-3 fatty acids total without supplements, g ^F^	0.8 (0.2, 1.5)						
Omega-6 fatty acids total, g ^G^	2.7 (-2.1, 7.6)					-0.03 (-0.1, 0.0) 0.025	
Omega -6 fatty acids without supplements, g ^H^	2.6 (-2.3, 7.5)					-0.03 (-0.1, 0.0) 0.023	
Dietary fiber, g	4.5 (1.3, 7.8)		-0.88 (-1.8, -0.01) 0.047		1.6 (0.05–3.1) ^I^ 0.044		
Carbohydrate, g	-93.1 (-129.4, -56.7)						0
Total sugar, g	38.0 (9.3, 66.7)					-0.005 (-0.009, 0.0) 0.048	
Natural sugar, g	-1.6 (-21.0, 17.8)		-0.16 (-0.3, -0.01) 0.031			-0.01 (-0.0, 0.1) 0.048	
Added sugar, g	-36.4 (-54.-18.9)						
Energy, kcal	-479 (606)				0.02 (0.01,0.03) ^I^ 0.010		
Alcohol, g	-4.7 (7.6)						

^A^ Mean and 95% confidence intervals (CI) for participants’ mean lipid profile changes between baseline and 12-month visits

^B^ Mean and CI for participants’ mean intake changes between baseline and 12-month visits

^C^ Statistically significant *p*-values for the association of intake mean changes and lipid profile mean changes

^D^ Sum of 20:5 (n-3) eicosapentaenoic acid and 22:6 (n-3) docosahexaenoic fatty acids from food and supplements

^E^ Sum of 18:3 (n-3) alpha-linolenic acid, 20:3 (n-3) eicosatrienoic acid, 20:5 (n-3) eicosapentaenoic acid, 22:3 (n-3) 13c,16c,19c-docasatrienoic fatty acid, 22:5 (n-3) docosapentaenoic acid, and 22:6 (n-3) docosahexaenoic acid from food and supplements

^F^ Sum of 18:3 (n-3) alpha linolenic acid, 20:3 (n-3) eicosatrienoic acid, 20:5 (n-3) eicosapentaenoic acid, 22:3 (n-3) 13c,16c,19c-docasatrienoic fatty acid, 22:5 (n-3) docosapentaenoic acid, and 22:6 (n-3) docosahexaenoic acid from food only

^G^ Sum of 18:2 (n-6) linoleic acid, 18:3 (n-6) gamma-linolenic acid, 20:2 (n-6) eicosadienoic acid, 20:3 (n-6) dihomo-gamma-linolenic acid, 20:4 (n-6) arachidonic acid, 22:2 (n-6) docosadienoic acid, and 22:4 (n-6) adrenic acid from food and supplements

^H^ Sum of 18:2 (n-6) linoleic acid, 18:3 (n-6) gamma-linolenic acid, 20:2 (n-6) eicosadienoic acid, 20:3 (n-6) dihomo-gamma-linolenic acid, 20:4 (n-6) arachidonic acid, 22:2 (n-6) docosadienoic acid, and 22:4 (n-6) adrenic acid from food only.

^I^ Adjusted for dependent variable baseline value.

### Association of changes in nutrient intake and changes in lipid profiles

The significant associations between changes in mean nutrient intake and changes in mean lipid profiles are presented in Table 3. Decreased TC levels were associated with decreased intake of total fat (p = 0.005). Decreased TG levels were associated with decreased intake of energy, total fat and trans-fat (all p ≤ 0.042) and with increased intake of dietary fiber (p = 0.044). Increased HDL-C levels were associated with increased intake of dietary fiber, and decreased intake of natural sugar (p ≤ 0.047). Decreased levels of TC-to-HDL-C ratio were associated with increased intake of polyunsaturated and omega-6 fatty acids and decreased intake of total sugar and natural sugar (all p ≤ 0.050).

Models were generated for the association of mean changes between nutrients and lipids with and without an adjustment for baseline dependent variables. The baseline adjustment only made a difference in the triglyceride mean change associations with dietary fiber and energy intake mean changes (see Table 3 footnote I).

### Associations between changes in lipid profile variables and changes in FSS

The decrease in mean FSS score was not associated with mean change in BMI from baseline to 12 months. Increased HDL-C (β = -0.05; 95% CI: -0.1, -0.0004; p = 0.048) was associated with greater decreases in FSS. Changes in TC (p = 0.005) were associated with greater decreases in FSS; 15/18 (83%) patients had both mean FSS decreases and mean TC decreases; three patients with decreases in FSS had increases in TC values but with increased HDL and LDL. Changes in LDL-C and TG were not associated with decreases in FSS.

In additional analyses, the associations between change in FSS over time and increase in HDL-C over time (all p ≤ 0.058) and TC (all p ≤ 0.007) persisted upon adjustment for average diet adherence, average servings of recommended foods, average servings of excluded foods and average exercise-NMES adherence.

## Discussion

The observed changes in nutrient intake on the study diet are consistent with changes expected from the guidelines to reduce or eliminate grains (carbohydrate) and dairy (carbohydrate and saturated fat), while encouraging large quantities of fiber-containing vegetables and fruits plus foods and supplements containing omega-3 fatty acids. The reductions in saturated fat, trans fat, added sugars, and increases in dietary fiber and omega-3 fatty acids are consistent with changes recommended in the Dietary Guidelines for Americans [25].

The study diet has similarities but also notable differences compared to the Paleolithic diet of Cordain et al. [27], which is higher in animal protein than the study diet, and excludes all grains, legumes and vegetable oils that were not available to pre-agricultural hominins. As a result, average protein intake on the study diet was lower (20.3 vs. 38% energy) whereas carbohydrate (37.3 vs 23% energy) and fat (43.8 vs 39% energy) were higher than the Paleolithic diet of Cordain et al. [27]. Average saturated fat intake was similar on both diets (8.6 vs 7% energy).

The study diet differs from the low-saturated fat Swank diet [28] and the plant-based, low-fat McDougall diet [7] in that it does not restrict consumption of animal protein, fat or saturated fat. The study diet and McDougall diet restrict dairy and eggs but the Swank diet allows <1% milk fat dairy and egg whites *ad libitum* and up to three whole eggs per week. The study diet and Swank diet allow fish and fish oil but the McDougall diet does not.

In previous work, we have shown that the study diet is associated with improvements on fatigue and also with improvements in secondary outcomes of walking performance, balance and quality of life [9, 29]. The principal focus of this research was to determine whether lipid profiles were associated with changes in fatigue outcomes [8–10]. BMI, TG, TC-to-HDL-C ratio and TG-to-HDL-C ratio all decreased and HDL-C increased over the 12-month study period. TC and HDL-C changes over 12 months were associated with changes in fatigue over 12 months.

Yadav et al. reported that HDL-C levels were similar in their very-low-fat, plant-based diet and waitlisted control Relapsing Remitting-MS groups and between the baseline and post-diet samples [7], whereas we observed an average 6.0 mg/dl increase in HDL-C over 12 months. In the Yadav et al. study, LDL-C decreased by 12 mg/dl and TC decreased by 13.2 mg/dl at 6 months in the diet group relative to controls. These decreases are similar to the average LDL-C and TC decreases of 10.4 mg/dl and 10.2 mg/dl, respectively, on the study diet at 12 months [7]. The decreases in BMI observed in both studies are not surprising because many diet interventions alter Caloric intake and induce changes to body weight and BMI. Yadav et al. found that 42.5% of the effect of the study diet on fatigue was attributable to weight loss [7]. In contrast, we did not find any associations between changes in BMI and change in FSS over time. However, individuals in the Yadav et al. study were on average overweight (BMI 28.4 ± 6.76, control group and 29.3 ± 7.42, diet group) while our participants were on average normal weight (BMI 24.5 ± 3.2 at baseline and 22.0 ± 2.2 at 12 months). Weight loss was not intended in our study, and neither drastic Caloric restriction nor fasting was recommended. Participants were advised to eat recommended foods until satiety.

The salient contrasting differences between the study diet and the very-low-fat, plant-based, diet [7] are the fat (44% vs. 14.4% energy) and carbohydrate (37% vs. ~76% energy) levels. Low-fat, high-carbohydrate diets tend to increase triglycerides and lower LDL-C [30]; they do not generally increase HDL-C. Studies have shown that Paleolithic diets, which tend to be lower in carbohydrate and added sugars, decrease TG and increase HDL-C compared to control diets [31, 32], which we also observed with the study diet. The changes in TG and HDL-C might be associated with the lower added sugar intake in the study diet. A cross-sectional study of NHANES data found that lower added sugar intake was associated with lower TG and higher HDL-C [33]. Evidence from a meta-analyses [12] supports the possibility that the decreases in TC, LDL-C, TG, TC-to-HDL-C ratio and TG-to-HDL-C ratio on the study diet may be associated with replacement of saturated fat with polyunsaturated and monounsaturated fats [12]. Dietary fat increases HDL-C by increasing apoA-I mRNA translation and decreasing its catabolism [34].

**Fig 4:**
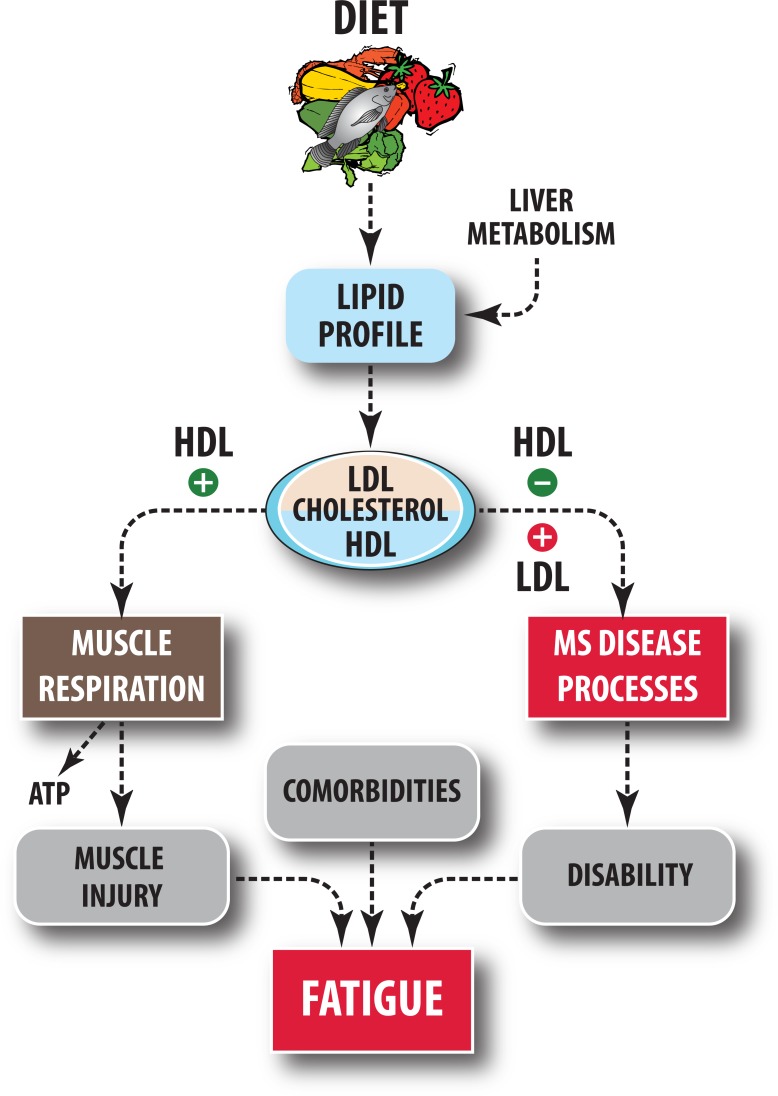
A schematic summarizing the hypothesized relationships between diet, lipid variables and MS fatigue. We hypothesize that diet-induced lipid changes can influence MS fatigue via effects on MS disease processes and muscle respiration. MS fatigue is associated with comorbidities, muscle injury and disability.

We hypothesize that skeletal muscle could be an important but overlooked organ for potential interactions between diet-induced lipid profile changes, metabolic activity and fatigue, because it is a key effector organ at which MS physical disability, fatigue and weakness manifest to patients. The critical role of cholesterol in muscle function is emerging in recent research [35]. HDL-C and its signature apolipoprotein, apoA-I, stimulate glucose uptake and increase respiration in muscle mitochondria [36]. In individuals 80 years and older, HDL-C is a reliable marker of frailty, physical performance, muscle strength, and functional status [37]. Fig 4 is a schematic that summarizes the hypothesized mechanisms that could plausibly mediate the effects of diet on fatigue. Diet modulates lipid profile, which in turn influences the MS disease process [17–21] and muscle function [35] resulting in benefits on MS fatigue.

Omega-3 fatty acids are potent modulators of systemic and central nervous system inflammation, neuronal membrane fluidity and neurotransmitter production [38]. There is evidence that omega-3 supplementation may have benefits on fatigue [39]. However, the randomized placebo-controlled studies of omega-3 supplementation on MS disease course have not yielded definitive results [40].

From our results, it is not possible to ascertain whether statins may be useful for the treatment of fatigue separately from diet. Statin treatment of hypercholesterolemia decreases LDL-C and TC prominently but the increases in HDL-C are more variable [41]. The Phase 2 MS-STAT trial of simvastatin in progressive MS yielded positive results on brain atrophy [42]; a follow-up replication trial is underway. Nonetheless, given that high 80 mg simvastatin doses were used in MS-STAT, the potential side effects of statins particularly muscle pain and life-threatening muscle damage must be considered.

One potential limitation of our study is that participants received a diet-based multimodal intervention that included dietary supplements, exercise, NMES, stress reduction, and varying amounts and types of dietary supplements. Our analyses indicated that the study diet component was likely the major contributing factor to BMI and lipid profile changes, because the changes in fatigue and body weight were associated with consumption of recommended foods. Another limitation is the use of a Food Frequency Questionnaire (FFQ) to estimate nutrient intake. FFQs are subject to bias, the questionnaire may not have included foods and supplements needed to adequately estimate the nutrient intake of the study diet, and respondents had to recall and average their food intake over the past year which is a cognitively difficult task [43]. Our study would be strengthened with more frequent FFQ administration and periodic administration of 24-hour dietary recalls, which elicits detailed diet information over the course of a day.

Another weakness of our study is the lack of a control diet group and adjustment for depression, which we acknowledge would have added strength to the study findings. Because of the lack of a control group, it is not possible to assess the contribution, if any, of the placebo effect and the observer or Hawthorne effect, in which the participants’ responses are altered as a result of study participation and observation rather than the intervention.

The positive results from this pilot study provide a strong scientific rationale for a larger controlled study. It may be necessary to consider a parallel group study design against another diet, such as the low saturated fat diet, to enable randomization and generate patient interest in study participation.

## Conclusions

The results from this pilot study of the diet-based multimodal intervention are consistent with the possibility that lipid profile biomarkers, particularly TC and HDL-C, may contribute to improvement in MS fatigue. Our results require confirmation given the limitations of the current pilot study design, which include the small sample size, lack of control group and randomization. However, if confirmed in larger studies, lipid monitoring may become useful for guiding fatigue treatment decisions.

## Supporting information

S1 Supplementary Data Information on supplements. (DOCX)

S2 Supplementary Data TREND checklist. (DOCX)

S3 Supplementary Data Study protocol. (DOCX)
