# Supplementary material for: Lipid profile is associated with decreased fatigue in individuals with progressive multiple sclerosis following a diet-based intervention: Results from a pilot study
Source: PLoS One. 2019 Jun 18;14(6):e0218075. doi: 10.1371/journal.pone.0218075 (PMC6581256; doi:10.1371/journal.pone.0218075)
Supplement: S1 Supplementary Data — (DOCX) [file pone.0218075.s001.docx]

**S1 SUPPLEMENTARY DATA**

**Lipid Profile is Associated with Decreased Fatigue in Individuals with Progressive Multiple Sclerosis Following a Diet-Based Intervention: Results from a Pilot Study**

Kelly Fellows, Terry Wahls, Richard W. Browne, Linda Rubenstein, Babita Bisht, Catherine A. Chenard, Linda Snetselaar, Bianca Weinstock-Guttman, Murali Ramanathan

**INFORMATION ON SUPPLEMENTS**

**Pinnaclife Full Spectrum** (Pinnaclife, Coralville, IA) contains:

Vitamin A 5000 IU (100% RDA), Vitamin D 180mg (300% RDA), D3 1050 IU (263%RDA), Vitamin E Mixed tocopherols and triols 50 IU (167% RDA), Vitamin K 20mcg (25% RDA), Thiamine 6 mg (353% RDA), Folic acid 800 mcg (200%), Vitamin B12 30mcg (500% RDA), Biotin 50 mcg (17%), Pantothenic acid 300 mg (300% RDA), Iodine 350 mcg (233% RDA), Zinc 30 mg (200%), Selenium 100 mcg (143% RDA), Copper 3 mg (150% RDA), Manganese 3 mg (150% RDA), Chromium 150 mcg (125%), Molybdenum 75 mcg (100% RDA), Boron 50 mcg, Nickel 5 mcg, Silicon 2 mg, Tin 10 mcg, Vanadium 10 mcg, Acetyl L-carnitine 50 mg, Coenzyme Q 10 50 mg, Alpha lipoic acid 50 mg, Lycopene 2 mg, Lutein 2 mg, Zeaxanthin 2 mg).

**Pinnaclife Essential** (Pinnaclife, Coralville, IA) contains:

An amino acid blend of Glycine, L Taurine, N-acetyl L-cysteine, Methylsulfonylmethane, Olive leaf extract (hydroxytyrosol 7 % standardized, L proline, Niacinamide, and Pyridoxine 2600 mg.

**Pinnaclife Mineral Boost** (Pinnaclife, Coralville, IA) contains:

Magnesium 500 mg, Calcium 400 mg plus Vitamin D3 750 IU.

**Pinnaclife Cleanse** (Pinnaclife, Coralville, IA) contains:

Digestive resistant Maltodextrose (glycemic index of 5) 10 grams soluble fiber, digestion resistant Maltodextrin 11.7 g and 25 mcg of a blend of Glycine, L-Taurine, N Acetylecysteine, Methylsulfonylmethane, Olive leaf extract (hydroxytyrosol 7% standardized), L Proline, Niacinamide and Pyridoxine) 25 mcg per scoop.
