# Supplementary material for: Lipid profile is associated with decreased fatigue in individuals with progressive multiple sclerosis following a diet-based intervention: Results from a pilot study
Source: PLoS One. 2019 Jun 18;14(6):e0218075. doi: 10.1371/journal.pone.0218075 (PMC6581256; doi:10.1371/journal.pone.0218075)
Supplement: S3 Supplementary Data — (DOCX) [file pone.0218075.s003.docx]

**Complete Research Protocol (HRP-503)**

Table of Contents

[Template Instructions 2](#_Toc440956501)

[1.0 Objectives 4](#_Toc440956502)

[2.0 Scientific Endpoints 4](#_Toc440956503)

[3.0 Background 4](#_Toc440956504)

[4.0 Study Design 4](#_Toc440956505)

[5.0 Local Number of Subjects 5](#_Toc440956506)

[6.0 Inclusion and Exclusion Criteria 5](#_Toc440956507)

[7.0 Vulnerable Populations 6](#_Toc440956508)

[8.0 Eligibility Screening 7](#_Toc440956509)

[9.0 Recruitment Methods 7](#_Toc440956510)

[10.0 Procedures Involved 8](#_Toc440956511)

[11.0 Study Timelines 9](#_Toc440956512)

[12.0 Setting 9](#_Toc440956513)

[13.0 Community-Based Participatory Research 10](#_Toc440956514)

[14.0 Resources and Qualifications 10](#_Toc440956515)

[15.0 Other Approvals 11](#_Toc440956516)

[16.0 Provisions to Protect the Privacy Interests of Subjects 11](#_Toc440956517)

[17.0 Data Management and Analysis 12](#_Toc440956518)

[18.0 Confidentiality 12](#_Toc440956519)

[A. Confidentiality of Study Data 13](#_Toc440956520)

[B. Confidentiality of Study Specimens 13](#_Toc440956521)

[19.0 Provisions to Monitor the Data to Ensure the Safety of Subjects 14](#_Toc440956522)

[20.0 Withdrawal of Subjects 15](#_Toc440956523)

[21.0 Risks to Subjects 16](#_Toc440956524)

[22.0 Potential Benefits to Subjects 16](#_Toc440956525)

[23.0 Compensation for Research-Related Injury 16](#_Toc440956526)

[24.0 Economic Burden to Subjects 17](#_Toc440956527)

[25.0 Compensation for Participation 17](#_Toc440956528)

[26.0 Consent Process 17](#_Toc440956529)

[27.0 Waiver or Alteration of Consent Process 21](#_Toc440956530)

[28.0 Process to Document Consent 22](#_Toc440956531)

[29.0 Multi-Site Research (Multisite/Multicenter Only) 23](#_Toc440956532)

[30.0 Banking Data or Specimens for Future Use 24](#_Toc440956533)

[31.0 Drugs or Devices 24](#_Toc440956534)

[32.0 Humanitarian Use Devices 25](#_Toc440956535)

**Template Instructions**

***Sections that do not apply:***

- In several sections, the addition of checkboxes for **Not Applicable** have been added to the template as responses.
  - If an N/A checkbox is present, select the appropriate justification from the list.
  - If an N/A checkbox is not present, or if none of the existing checkboxes apply to your study, you must write in your own justification.
- In addition:
  - For research where the only study procedures are records/chart review: Sections 19, 20, 22, 23, 24, 25, 31, and 32 do not apply.
  - For exempt research: Sections 31 and 32 do not apply.

**Studies with multiple participant groups:**

- If this study involves multiple participant groups (e.g. parents and children), provide information in applicable sections for each participant group. Clearly label responses when they differ. For example:

Response:

Intervention Group:

Control Group:

**Formatting:**

- Do not remove template instructions or section headings when they do not apply to your study.

If you are pasting information from other documents using the “Merge Formatting” Paste option will maintain the formatting of the response boxes.

**Amendments:**

- When making modifications or revisions to this and other documents, use the **Track Changes** function in Microsoft Word.
- Update the version date or number **on Page 3.**

**PROTOCOL TITLE:**

*Include the full protocol title.*

Response:

Cholesterol Biomarkers and Oxysterols in Multiple Sclerosis Fatigue

**PRINCIPAL INVESTIGATOR:**

*Name*

*Department*

*Telephone Number*

*Email Address*

Response:

Murali Ramanathan

Pharmaceutical Sciences

716-645-4846

Murali@Buffalo.Edu

**VERSION:**

*Include the version date or number.*

Response:

2017-119

**GRANT APPLICABILITY:**

*Indicate whether this protocol is funded by a grant (e.g. NIH, foundation grant). For a grant with multiple aims, indicate which aims are covered by this research proposal.*


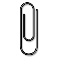
*NOTE: This question does not apply to studies funded by a sponsor contract.*

*Include a copy of the grant proposal with your submission.*

Response:

This project has is not funded. Grant applications are planned after the preliminary analyses are conducted.

RESEARCH REPOSITORY:

*Indicate where the research files will be kept, including when the study has been closed. The repository should include, at minimum, copies of IRB correspondence (approval, determination letters) as well as signed consent documents. This documentation should be maintained for 3 years after the study has been closed.*

Response:

Location: Kapoor 355

Address: South Campus, University at Buffalo

Department: Pharmaceutical Sciences

No patient recruitment – signed consents not applicable.

# Objectives

- 1. Describe the purpose, specific aims, or objectives of this research.

Response:

Fatigue is a very frequent MS symptom that affects greater >75% of patients [^1-3^](#_ENREF_1) [^4^](#_ENREF_4). Fatigue adversely affects MS patients’ quality of life and activities of daily living [^2^](#_ENREF_2)^,^ [^5^](#_ENREF_5). Fatigue worsens with MS disease progression and makes it more difficult for people with MS to adapt to their chronic disease [^6^](#_ENREF_6).This Supplementary Study will build on the important pioneering work by Dr. Terry Wahls on the modified Paleolithic diet in MS, which is known as the Wahls’ Elimination Diet. It will leverage clinical samples collected in the course of Dr. Wahls’ currently funded National Multiple Sclerosis Society-funded clinical study to assess the role of metabolism in fatigue and from the smaller longitudinal study (n = 20) of multimodal interventions (combination of Paleolithic diet with supplements plus stretching, strengthening, meditation and massage) to reduce fatigue. We will compare the effects of Usual Diet vs. Swank Diet vs. Wahls’ Elimination Diet on fatigue in MS patients. We will compare baseline vs. 12 month follow-up samples ( in the multimodal study.

The cholesterol pathway is important for immune responses and neuronal functions in the central nervous system. The aims of this clinical study are to delineate the molecular mechanisms and to characterize the relationships between the cholesterol pathway and fatigue in multiple sclerosis (MS).

From our research, there is compelling evidence that adverse cholesterol profiles are associated with MS disease progression. The pathophysiological mechanisms mediating these adverse associations in MS are not known. Our strategy for dissecting the underlying pathophysiological mechanisms is to target molecular mediators of the inter-dependence between CNS and peripheral cholesterol homeostasis in MS patients. Based on our results, we hypothesize that the associations between dietary changes and fatigue are mediated by metabolic changes that are reflected in serum cholesterol serum cholesterol and are driven by key contributions from oxysterols, which are a promising class of endogenous cholesterol metabolites and signaling molecules capable of: i) crossing the blood brain barrier (BBB) and, ii) regulating the homeostasis between CNS and peripheral cholesterol.

We will systematically investigate the oxysterols 24-hydroxycholesterol (24HC), 27-hydroxycholesterol (27HC), 7α-hydroxycholesterol (7αHC) and 7-ketocholesterol (7KC) because they are potent transcription factor ligands and regulatory mediators of cholesterol and immune homeostasis. These oxysterols have been shown to modulate the interactions between immune cells and the vascular cells of the BBB and CNS pathophysiology.

**Specific Aim 1 >** Characterize the longitudinal changes in lipid profile occuring during the “Usual Diet Phase” and in the Swank Diet and Wahls’ Elimination Diet groups.

For this aim, a comprehensive lipid profile will be obtained. The profile will consist of: i) overall lipid profile, ii) serum free fatty acid and lipoprotein cholesteryl ester fatty acid profile, iii) apolipoprotein profile, iv) serum paraoxonase activities, serum paraoxonase genotype and C-reactive protein level, and v) serum oxysterol profile.

**Specific Aim 2 >** Investigate the associations if any, between the observed changes in lipid profile in Aim 1 and fatigue outcomes. Identify the lipid profile biomarkers that are most strongly associated with fatigue outcomes in MS. Assess whether these lipid profiles changes are potential mediators of the effects of diet on fatigue.

When successfully completed, this project will identify and characterize important molecular mechanisms that could explain the pathophysiological impact of the cholesterol pathway on MS fatigue. The findings can be leveraged for targeting therapeutic and lifestyle-based intervention strategies to reduce fatigue in MS patients.

- 1. State the hypotheses to be tested, if applicable.

NOTE: A hypothesis is a specific, testable prediction about what you expect to happen in your study that corresponds with your above listed objectives.

Response:

**Hypothesis 1 >** The Wahls’ Elimination Diet is associated with significant improvements in the lipid profile including decreased total cholesterol, decreased low density cholesterol (LDL-C) and apolipoprotein B (ApoB) and increases in high density cholesterol (HDL-C), decreases in cholesteryl esters conjugated with saturated fatty acids and decreased CRP levels. The Wahls’ Elimination diet increases 24-hydroxycholesterol (24HC) and decreases 7-ketocholesterol (7KC) levels.

**Hypothesis 2 >** The improvements in the lipid profile are mediators of benefits of the Wahls’ Elimination Diet on fatigue. Subjects with greater improvements in their lipid profile will exhibit greater improvements in fatigue outcomes.

# Scientific Endpoints

*2.1* Describe *the scientific endpoint(s), the main result or occurrence under study.*

*NOTE: Scientific endpoints are outcomes defined before the study begins to determine whether the objectives of the study have been met and to draw conclusions from the data. Include primary and secondary endpoints. Some example endpoints are: reduction of symptoms, improvement in quality of life, or survival. Your response should* ***not*** *be a date.*

Response:

The primary goals are to study the associations of cholesterol biomarkers and oxysterol profiles with quantitative fatigue measures obtained in the Wahls study.

# Background

- 1. Provide the scientific or scholarly background, rationale, and significance of the research based on the existing literature and how it will contribute to existing knowledge. Describe any gaps in current knowledge. Include relevant preliminary findings or prior research by the investigator.

Response:

The cholesterol pathway is important for immune responses and neuronal functions in the central nervous system. The aims of this clinical study are to delineate the molecular mechanisms and to characterize the relationships between the cholesterol pathway and fatigue in multiple sclerosis (MS). From our research, there is compelling evidence that adverse cholesterol profiles are associated with MS disease progression. Our proposed strategy is to investigate the role of cholesterol biomarkers in fatigue.

- 1. Include complete citations or references.

Response:

The publications from our previous work are listed below:

1. Weinstock-Guttman B, et al. Lipid profiles are associated with lesion formation over 24 months in interferon-beta treated patients following the first demyelinating event. Journal of Neurology, Neurosurgery and Psychiatry. 84: 1186-1191 (2013). **This earliest report, based on clinically derived lipid profiles demonstrated that LDL-C and TC levels are associated with inflammatory MRI activity measures.**
2. Weinstock-Guttman B, et al. Interactions of serum cholesterol with anti-herpesvirus responses affect disease progression in clinically isolated syndromes. Journal of Neuroimmunology. 263: 121-127 (2013). **We investigated whether there was statistical evidence for interactions between the cholesterol pathway and anti-EBV antibody status, a known risk factor for MS susceptibility and progression.**
3. Browne RW, et al. Apolipoproteins are associated with new MRI lesions and deep gray matter atrophy in clinically isolated syndromes. Journal of Neurology, Neurosurgery and Psychiatry. 85(8):859-64 (2014). Reports results from apolipoproteins and other cholesterol biomarkers with MRI measures in MS. **This report was based on lipid, lipoprotein, cholesteryl ester and apolipoprotein profiles analyzed at Buffalo for all SET study sites. The results corroborated the findings from our first paper.**
4. Browne RW et al. Serum lipoprotein composition and vitamin D metabolite levels in clinically isolated syndromes: Results from a multi-center study. Journal of Steroid Biochemistry and Molecular Biology. 143C: 424-433 (2014). **Reports results from genetics and demonstrates the associations of cholesterol biomarkers and candidate genetic variations with vitamin D metabolites in MS.**
5. Kappus N et al. Cardiovascular risk factors are associated with increased lesion burden and brain atrophy in multiple sclerosis. Journal of Neurology, Neurosurgery and Psychiatry (2015). **We investigated cardiovascular risk factors including hypertension, heart problems, type 1 diabetes, smoking and overweight/obesity. MS patients with one or more risks factors showed increased lesion burden and more advanced gray matter atrophy.**
6. Narayanaswamy R et al. Simultaneous determination of oxysterols, cholesterol and 25-hydroxy-vitamin D3 in human plasma by LC-UV-MS. PLOS One 10(4): e0123771 (2015). **Summarizes validation of and innovations to our oxysterol assay. Provides preliminary results for oxysterols in MS.**
7. Fellow K et al. Protective associations of HDL with blood brain barrier injury in multiple sclerosis patients. Journal of Lipid Research (2015). **Demonstrates that high HDL is associated with lower BBB breakdown**.

**REFERENCES GENERAL**

1. Dendrou CA, Fugger L, Friese MA. Immunopathology of multiple sclerosis. Nature reviews Immunology 2015; **15**(9): 545-58.

2. Mahad DH, Trapp BD, Lassmann H. Pathological mechanisms in progressive multiple sclerosis. The Lancet Neurology 2015; **14**(2): 183-93.

3. Feinstein A, Freeman J, Lo AC. Treatment of progressive multiple sclerosis: what works, what does not, and what is needed. The Lancet Neurology 2015; **14**(2): 194-207.

4. Haghikia A, Hohlfeld R, Gold R, Fugger L. Therapies for multiple sclerosis: translational achievements and outstanding needs. Trends in molecular medicine 2013; **19**(5): 309-19.

5. Weinstock-Guttman B, Zivadinov R, Mahfooz N, et al. Serum lipid profiles are associated with disability and MRI outcomes in multiple sclerosis. Journal of neuroinflammation 2011; **8**: 127.

6. Browne RW, Weinstock-Guttman B, Horakova D, et al. Apolipoproteins are associated with new MRI lesions and deep grey matter atrophy in clinically isolated syndromes. Journal of neurology, neurosurgery, and psychiatry 2014.

7. Tettey P, Simpson S, Jr., Taylor B, et al. An adverse lipid profile is associated with disability and progression in disability, in people with MS. Mult Scler 2014; **20**(13): 1737-44.

8. Tettey P, Simpson S, Jr., Taylor B, et al. Adverse lipid profile is not associated with relapse risk in MS: results from an observational cohort study. J Neurol Sci 2014; **340**(1-2): 230-2.

9. Tettey P, Simpson S, Jr., Taylor BV, van der Mei IA. Vascular comorbidities in the onset and progression of multiple sclerosis. J Neurol Sci 2014; **347**(1-2): 23-33.

10. Weinstock-Guttman B, Zivadinov R, Ramanathan M. Inter-dependence of vitamin D levels with serum lipid profiles in multiple sclerosis. J Neurol Sci 2011.

11. Ascherio A, Munger KL. Environmental risk factors for multiple sclerosis. Part II: Noninfectious factors. Ann Neurol 2007; **61**(6): 504-13.

12. Kantarci O, Wingerchuk D. Epidemiology and natural history of multiple sclerosis: new insights. Curr Opin Neurol 2006; **19**(3): 248-54.

13. Marrie RA, Rudick R, Horwitz R, et al. Vascular comorbidity is associated with more rapid disability progression in multiple sclerosis. Neurology 2010; **74**(13): 1041-7.

14. Giubilei F, Antonini G, Di Legge S, et al. Blood cholesterol and MRI activity in first clinical episode suggestive of multiple sclerosis. Acta Neurol Scand 2002; **106**(2): 109-12.

15. Jamroz-Wisniewska A, Beltowski J, Stelmasiak Z, Bartosik-Psujek H. Paraoxonase 1 activity in different types of multiple sclerosis. Mult Scler 2009; **15**(3): 399-402.

16. Meyers L, Groover CJ, Douglas J, et al. A role for Apolipoprotein A-I in the pathogenesis of multiple sclerosis. Journal of neuroimmunology 2014; **277**(1-2): 176-85.

17. Shin S, Walz KA, Archambault AS, et al. Apolipoprotein E mediation of neuro-inflammation in a murine model of multiple sclerosis. Journal of neuroimmunology 2014; **271**(1-2): 8-17.

18. Alexander JS, Harris MK, Wells SR, et al. Alterations in serum MMP-8, MMP-9, IL-12p40 and IL-23 in multiple sclerosis patients treated with interferon-beta1b. Mult Scler 2010; **16**(7): 801-9.

19. Alexander JS, Zivadinov R, Maghzi AH, Ganta VC, Harris MK, Minagar A. Multiple sclerosis and cerebral endothelial dysfunction: Mechanisms. Pathophysiology 2010.

20. Minagar A, Carpenter A, Alexander JS. The destructive alliance: interactions of leukocytes, cerebral endothelial cells, and the immune cascade in pathogenesis of multiple sclerosis. Int Rev Neurobiol 2007; **79**: 1-11.

21. Minagar A, Jy W, Jimenez JJ, Alexander JS. Multiple sclerosis as a vascular disease. Neurol Res 2006; **28**(3): 230-5.

22. Poli G, Sottero B, Gargiulo S, Leonarduzzi G. Cholesterol oxidation products in the vascular remodeling due to atherosclerosis. Molecular Aspects of Medicine 2009; **30**(3): 180-9.

23. Lyons MA, Brown AJ. 7-Ketocholesterol. Int J Biochem Cell Biol 1999; **31**(3-4): 369-75.

24. Poli G, Biasi F, Leonarduzzi G. Oxysterols in the pathogenesis of major chronic diseases. Redox Biology 2013; **1**(1): 125-30.

25. Lemaire-Ewing S, Prunet C, Montange T, et al. Comparison of the cytotoxic, pro-oxidant and pro-inflammatory characteristics of different oxysterols. Cell biology and toxicology 2005; **21**(2): 97-114.

26. Trousson A, Bernard S, Petit PX, et al. 25-hydroxycholesterol provokes oligodendrocyte cell line apoptosis and stimulates the secreted phospholipase A2 type IIA via LXR beta and PXR. Journal of neurochemistry 2009; **109**(4): 945-58.

27. Gargiulo S, Sottero B, Gamba P, Chiarpotto E, Poli G, Leonarduzzi G. Plaque oxysterols induce unbalanced up-regulation of matrix metalloproteinase-9 in macrophagic cells through redox-sensitive signaling pathways: Implications regarding the vulnerability of atherosclerotic lesions. Free Radic Biol Med 2011; **51**(4): 844-55.

28. Glass CK, Saijo K. Immunology: Oxysterols hold T cells in check. Nature 2008; **455**(7209): 40-1.

29. Okabe A, Urano Y, Itoh S, et al. Adaptive responses induced by 24S-hydroxycholesterol through liver X receptor pathway reduce 7-ketocholesterol-caused neuronal cell death. Redox Biol 2013; **2**: 28-35.

30. Chen ZH, Saito Y, Yoshida Y, Sekine A, Noguchi N, Niki E. 4-Hydroxynonenal induces adaptive response and enhances PC12 cell tolerance primarily through induction of thioredoxin reductase 1 via activation of Nrf2. J Biol Chem 2005; **280**(51): 41921-7.

31. Roxburgh RH, Seaman SR, Masterman T, et al. Multiple Sclerosis Severity Score: using disability and disease duration to rate disease severity. Neurology 2005; **64**(7): 1144-51.

32. Anonymous. Third Report of the Expert Panel on Detection, Evaluation, and Treatment of High Blood Cholesterol in Adults (Adult Treatment Panel III). 2001. <http://www.nhlbi.nih.gov/guidelines/cholesterol/atglance.pdf>.

# Study Design

- 1. Describe and explain the study design (e.g. case-control, cross-sectional, ethnographic, experimental, interventional, longitudinal, observational).

Response:

Our study will analyze blood samples from Dr. Wahls’ prospective study and her completed multimodal study.

Dr. Wahls’ study has a prospective, longitudinal, 36-week-long, randomized, parallel group study design in which MS patients will be randomized from a lead-in “Usual Diet Phase” to either the Swank Diet or the Wahls’ Elimination Diet.

The primary outcome is fatigue as measured on the Fatigue Severity Scale and Modified Fatigue Impact Scale.

The study has 4 visits over 36 weeks with each visit being 12 weeks apart. There will be a 12-week-long lead-in “Usual Diet Phase” to assess the ability of enrolled subjects to complete daily food records and collect fatigue and other health data while participants are consuming their usual diet.

The subjects who satisfactorily complete food records at Visit 1 will continue into the Intervention phase of the study at Visit 2 (Week 12) and be randomized to either the Swank Diet or the Wahls’ Elimination Diet.

The subjects and their adult companion (if present) will be trained at Visit 2 on the relevant study diet by a Registered Dietitian Nutritionist who will then call the subject weekly for 4 weeks to provide coaching and support for adhering to the study diet. The final coaching call will occur at Month 2.

The subject will return at Week 24 (Visit 3) and Week 36 (Visit 4). Dietary assessments will include a food frequency questionnaire and a 24-hour weighed food records. The assessments will include motor assessments, symptom questionnaires and dietary assessments.

Motor assessments will include a 6-minute walk test, 25-foot walk test, 9-hole peg board to test hand function and symbol digit test to testing cognitive function. Questionnaires to assess fatigue, quality of life and medical diagnoses are also included in the assessments. The assessments will be completed on or around each study visit.

The longitudinal multimodal study is already completed. The intervention investigated to reduce fatigue in this study was multimodal (combination of Paleolithic diet with supplements plus stretching, strengthening, meditation and massage).

# Local Number of Subjects

- 1. Indicate the total number of subjects that will be enrolled or records that will be reviewed locally.

Response:

No recruitment or enrollment proposed by the Buffalo investigators.

- 1. If applicable, indicate how many subjects you expect to screen to reach your target sample (i.e. your screen failure rate).

Response:

No screening, recruitment.

- 1. Justify the feasibility of recruiting the proposed number of eligible subjects within the anticipated recruitment period. For example, how many potential subjects do you have access to? What percentage of those potential subjects do you need to recruit?

Response:

No recruitment.

# Inclusion and Exclusion Criteria

- 1. Describe the criteria that define who will be **included** in your final study sample.

NOTE: This may be done in bullet point fashion.

Response:

Not applicable. No patient recruitment is proposed. This is analysis of samples that will be collected by Dr. Terry Wahls’ group at the University of Iowa.

- 1. Describe the criteria that define who will be **excluded** from your final study sample.

NOTE: This may be done in bullet point fashion.

Response:

Not applicable. No patient recruitment is proposed. This is analysis of samples that will be collected by Dr. Terry Wahls’ group in Iowa

- 1. Indicate specifically whether you will include any of the following special populations in your study using the checkboxes below.

**NOTE: Members of special populations may not be targeted for enrollment in your study unless you indicate this in your inclusion criteria.**

Response:

Adults unable to consent

Individuals who are not yet adults (infants, children, teenagers)

Pregnant women

Prisoners

- 1. Indicate whether you will include non-English speaking individuals in your study. **Provide justification if you will exclude non-English speaking individuals.**

In order to meet one of the primary ethical principles of equitable selection of subjects, non-English speaking individuals may **not** be routinely excluded from research as a matter of convenience.

In cases where the research is of therapeutic intent or is designed to investigate areas that would necessarily require certain populations who may not speak English, the researcher is required to make efforts to recruit and include non-English speaking individuals. However, there are studies in which it would be reasonable to limit subjects to those who speak English. Some examples include pilot studies, small unfunded studies with validated instruments not available in other languages, studies with numerous questionnaires, and some non-therapeutic studies which offer no direct benefit.

Response:

No recruitment

# Vulnerable Populations

If the research involves special populations that are considered vulnerable, **describe the safeguards included to protect their rights and welfare.**

NOTE: You should refer to the appropriate checklists, referenced below, to ensure you have provided adequate detail regarding safeguards and protections. You do not, however, need to provide these checklists to the IRB.

- 1. For research that involves **pregnant women,** safeguards include:
     NOTE CHECKLIST: Pregnant Women (HRP-412)

Response:

✔**N/A**: This research does not involve pregnant women.

- 1. For research that involves **neonates of uncertain viability or non-viable neonates,** safeguards include:

NOTE CHECKLISTS: Non-Viable Neonates (HRP-413), or Neonates of Uncertain Viability (HRP-414)

Response:

✔ **N/A:** This research does not involve non-viable neonates or neonates of uncertain viability.

- 1. For research that involves **prisoners**, safeguards include:

NOTE CHECKLIST: Prisoners (HRP-415)

Response:

✔ **N/A:** This research does not involve prisoners.

- 1. For research that involves **persons who have not attained the legal age for consent to treatments or procedures involved in the research (“children”)**, safeguards include:
     NOTE CHECKLIST: Children (HRP-416)

Response:

✔ **N/A:** This research does not involve persons who have not attained the legal age for consent to treatments or procedures (“children”).

- 1. For research that involves **cognitively impaired adults**, safeguards include:

NOTE CHECKLIST: Cognitively Impaired Adults (HRP-417)

Response:

✔ **N/A:** This research does not involve cognitively impaired adults.

- 1. Consider if other specifically targeted populations such as students, employees of a specific firm, or educationally or economically disadvantaged persons are vulnerable. **Provide information regarding their safeguards and protections, including safeguards to eliminate coercion or undue influence.**

Response:

# Eligibility Screening

- 1. Describe **screening procedures** for determining subjects’ eligibility. Screening refers to determining if prospective participants meet inclusion and exclusion criteria.


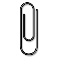
Include all relevant screening documents with your submission (e.g. screening protocol, script, questionnaire).

Response:

✔ **N/A:** There is no screening as part of this protocol.

# Recruitment Methods

**N/A:** This is a records review only, and subjects will not be recruited. NOTE: If you select this option, please make sure that all records review procedures and inclusion/exclusion screening are adequately described in other sections.

- 1. Describe when, where, and how potential subjects will be recruited.

NOTE: Recruitment refers to how you are identifying potential participants and introducing them to the study. Include specific methods you will use (e.g. searching charts for specific ICD code numbers, Research Participant Groups, posted advertisements, etc.).

Response:

Not applicable. No recruitment as part of this protocol.

- 1. Describe how you will protect the privacy interests of prospective subjects during the recruitment process.

NOTE: Privacy refers to an individual’s right to control access to him or herself.

Response:

Not applicable. No recruitment.

- 1. Identify any materials that will be used to recruit subjects.

NOTE: Examples include scripts for telephone calls, in person announcements / presentations, email invitations.


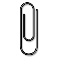
For advertisements, include the final copy of printed advertisements with your submission. When advertisements are taped for broadcast, attach the final audio/video tape. NOTE: You may submit the wording of the advertisement prior to taping to ensure there will be no IRB-required revisions, provided the IRB also reviews and approves the final version.

Response:

Not applicable. No recruitment materials.

# Procedures Involved

- 1. Provide a description of **all research procedures or activities** being performed and when they are performed once a subject is screened and determined to be eligible. Provide as much detail as possible.

NOTE: This should serve as a blueprint for your study and include enough detail so that another investigator could pick up your protocol and replicate the research. For studies that have multiple or complex visits or procedures, consider the addition of a schedule of events table in in your response.

Response:

No procedures will be conducted as part of this study. All recruitment, procedures, interventions sample collection are being conducted by Dr. Wahls’ team at the University at Iowa. The samples and data collection from the first study is complete and the second study is ongoing. Patients have provided informed consent for use of the data and samples for research for both studies to Dr. Wahls’ team at the University at Iowa. We will receive the samples by overnight delivery (e.g., UPS, FedEx etc.) on dry ice.

- 1. Describe what data will be collected.

NOTE: For studies with multiple data collection points or long-term follow up, consider the addition of a schedule or table in your response.

Response:

No procedures.

- 1. List any instruments or measurement tools used to collect data (e.g. questionnaire, interview guide, validated instrument, data collection form).

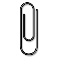

     Include copies of these documents with your submission.

Response:

No interventions or procedures on patients for this project. Previously collected blood samples will be analyzed using instruments in Dr. Browne’s laboratory.

- 1. Describe any source records that will be used to collect data about subjects (e.g. school records, electronic medical records).

Response:

This is analysis of samples and data that will be collected by Dr. Terry Wahls’ group in Iowa. We will receive clinical, demographic and fatigue outcome data from our Iowa collaborators. The Buffalo investigators will receive only de-identified samples. Identifiers are not needed for our study. Dr. Wahls and her medical team at Iowa have identifiers but we will work with de-identified data. Raw charts will not be received. Relevant data from the charts will be summarized in files by Dr. Wahls’ team. The de-identified data will be received electronically.

- 1. Indicate whether or not **individual** subject results, such as results of investigational diagnostic tests, genetic tests, or incidental findings will be shared with subjects or others (e.g., the subject’s primary care physician) and if so, describe how these will be shared.

Response:

The individual subjects results will not be shared. Aggregate statistical findings will be published in peer-reviewed literature and professional conferences.

- 1. Indicate whether or not **study** results will be shared with subjects or others, and if so, describe how these will be shared.

Response:

Study results will be published in the literature and shared with sponsors/funding agencies. Results may be shared with qualified scientists involved in MS research as part of research collaborations.

# Study Timelines

- 1. Describe the anticipated duration needed to enroll all study subjects.

Response:

No enrollment.

- 1. Describe the duration of an individual subject’s participation in the study. Include length of study visits, and overall study follow-up time.

Response:

No enrollment.

- 1. Describe the estimated duration for the investigators to complete this study (i.e. all data is collected and all analyses have been completed).

Response:

Approximately 5 years. Data acquisition will require 3 years and analysis will require 2 additional years.

# Setting

- 1. Describe all facilities/sites where you will be conducting research procedures. Include a description of the security and privacy of the facilities (e.g. locked facility, limited access, privacy barriers). Facility, department, and type of room are relevant. Do not abbreviate facility names.

NOTE: Examples of acceptable response may be: “A classroom setting in the Department of Psychology equipped with a computer with relevant survey administration software,” “The angiogram suite at Buffalo General Medical Center, a fully accredited tertiary care institution within New York State with badge access,” or, “Community Center meeting hall.”

Response:

A University research laboratory. All rooms have barriers and locks.

- 1. For research conducted outside of UB and its affiliates, describe:
- Site-specific regulations or customs affecting the research
- Local scientific and ethical review structure

NOTE: This question is referring to UB affiliated research taking place outside UB, i.e. research conducted in the community, school-based research, international research, etc. It is not referring to multi-site research. UB affiliated institutions include Kaleida Health, ECMC, and Roswell Park Cancer Institute.

Response:

✔ **N/A:** This study is not conducted outside of UB or its affiliates.

# Community-Based Participatory Research

- 1. Describe involvement of the community in the design and conduct of the research.

NOTE: Community-Based Participatory Research (CBPR) is a collaborative approach to research that equitably involves all partners in the research process and recognizes the unique strengths that each brings. CBPR begins with a research topic of importance to the community, has the aim of combining knowledge with action and achieving social change to improve health outcomes and eliminate health disparities.

Response:

✔ **N/A:** This study does not utilize CBPR.

- 1. Describe the composition and involvement of a community advisory board.

Response:

✔ **N/A:** This study does not have a community advisory board.

# Resources and Qualifications

- 1. Describe the qualifications (e.g., education, training, experience, expertise, or certifications) of the Principal Investigator **and** staff to perform the research. When applicable describe their knowledge of the local study sites, culture, and society. Provide enough information to convince the IRB that you have qualified staff for the proposed research.

NOTE: If you specify a person by name, a change to that person will require prior approval by the IRB. If you specify a person by role (e.g., coordinator, research assistant, co-investigator, or pharmacist), a change to that person will not usually require prior approval by the IRB, provided that the person meets the qualifications described to fulfill their roles.

Response:

Dr. Ramanathan, Ph.D., is Professor of Pharmaceutical Sciences and Neurology and has extensive experience in MS research and in the role of cholesterol in MS disease progression. He has the necessary multi-disciplinary expertise and experience with coordinating research involving numerous investigators with diverse backgrounds that are critical for coordination and enabling the success of the project. He has an extensive collaborative track record and joint publications with Dr. Weinstock-Guttman and Browne.

Dr. Richard Browne, Ph.D., will be responsible for the mass spectrometric and lipid analysis aspects of the project. The Co-Investigator has expertise in high sensitivity liquid chromatography mass spectrometry that is necessary for specific and selective quantitation of the cholesterol and oxysterols for the project.

Dr. Bianca Weinstock-Guttman, M.D., is the Director of the Baird Multiple Sclerosis Center at the Department of Neurology at the University at Buffalo. Dr. Weinstock-Guttman’s recent work has focused on genetic susceptibility and environmental factors, including the role of cholesterol in understanding MS disease progression and treatment outcomes.

**Describe other resources available to conduct the research.**

- 1. Describe the time and effort that the Principal Investigator and research staff will devote to conducting and completing the research.

NOTE: Examples include the percentage of Full Time Equivalents (FTE), hours per week. The question will elicit whether there are appropriate resources to conduct the research.

Response:

Drs. Ramanathan and Browne will each devote 0.5 calendar months per year. Drs. Weinstock-Guttman will devote 0.25 calendar months per year.

- 1. Describe the availability of medical or psychological resources that subjects might need as a result of anticipated consequences of the human research, if applicable.

NOTE: One example includes: on-call availability of a counselor or psychologist for a study that screens subjects for depression.

Response:

No recruitment.

- 1. Describe your process to ensure that all persons assisting with the research are adequately informed about the protocol, the research procedures, and their duties and functions.

Response:

No recruitment. The project proposal has been shared with all the investigators. The project proposal will be shared with all persons assisting with the research when the project is funded. Their immediate supervisors will inform them of their duties and functions.

# Other Approvals

- 1. Describe any approvals that will be obtained prior to commencing the research (e.g., school, external site, funding agency, laboratory, radiation safety, or biosafety).

Response:

✔ **N/A:** This study does not require any other approvals.

# Provisions to Protect the Privacy Interests of Subjects

- 1. Describe how you will protect subjects’ privacy interests during the course of this research.

NOTE: Privacy refers to an individual’s right to control access to him or herself. Privacy applies to the person. Confidentiality refers to how data collected about individuals for the research will be protected by the researcher from release. Confidentiality applies to the data.

Examples of appropriate responses include: “participant only meets with a study coordinator in a classroom setting where no one can overhear”, or “the participant is reminded that they are free to refuse to answer any questions that they do not feel comfortable answering.”

Response:

- 1. Indicate how the research team is permitted to access any sources of information about the subjects.

NOTE: Examples of appropriate responses include: school permission for review of records, consent of the subject, HIPAA waiver. This question **does apply** to records reviews.

Response:

# Data Management and Analysis

- 1. Describe the data analysis plan, including any statistical procedures. This section applies to both quantitative and qualitative analysis.

Response:

The demographic, clinical and biomarker data will be centrally organized in a relational database (e.g., Access) and exported for statistical analysis. Range and consistency checks will be performed to identify errors. SPSS Statistics (IBM) will be used. We will assess descriptive statistics, distributions and pairwise correlations to inform analyses. A systematic hierarchical approach to analysis will be employed given the number of oxysterols, clinical and fatigue measures. Analyses will correct for multiple testing using the Benjamini-Hochberg method [20](#_ENREF_20).

Where necessary, clinical thresholds from the National Cholesterol Education Program Guidelines [32](#_ENREF_32) of HDL < 40, LDL-C ≥ 130, and TC ≥ 200 mg/dl will be used to create indicator variables for classifying subjects with low HDL-C, high LDL-C and high TC, respectively; hypercholesterolemia will be defined as TC ≥ 240 mg/dl. Appropriate regression analyses will be used for assessing associations of cholesterol biomarker and the clinical and fatigue variables (dependent variables). Count variables, e.g., relapse rate, will be analyzed with negative binomial regression. Ordinal regression will be used for EDSS.

Several clinically relevant questions will be addressed in the analyses. We expect the cholesterol profiles to differ between the Usual Diet phase vs. Swank Diet vs. Wahls’ Elimination Diet groups. The effects of diet on individual apolipoproteins, CRP and paraoxonase and other markers will also be investigated. Likewise, we will investigate whether oxysterol levels differ between the Usual Diet, Swank Diet and Wahls’ Diet groups? Given the longitudinal design we will also be able identify those oxysterols whose changes are associated with changes in lipid profile variables.

Since we hypothesize that decreased 24HCand increased 7KC are associated with greater neurodegeneration in MS, our primary analysis will evaluate the associations of changes in these oxysterols in the Usual Diet, Swank Diet and Wahls’ Elimination Diet groups. The interpretation framework was noted previously: 24HC is a marker of CNS cholesterol metabolism and 7KC is a marker of oxidative stress.

We will generally adopt the statistical framework and strategies used by Wahls’ et al. in their primary outcome analyses wherever appropriate. We have data on several key covariates that may require consideration. The repeated measures regression analyses will adjust for age and sex because cholesterolemia risk increases with age and is more frequent in males. We will also explore other covariate adjustments. For example, cholesterol profiles depend on body mass index (BMI), which we will adjust for.

- 1. If applicable, provide a power analysis.

NOTE: This may not apply to certain types of studies, including chart/records reviews, survey studies, or observational studies. This question is asked to elicit whether the investigator has an adequate sample size to achieve the study objectives and justify a conclusion.

Response:

Not applicable. No patient recruitment is planned.

- 1. Describe any procedures that will be used for quality control of collected data.

Response:

The inter-day and intra-day CVs for cholesterol biomarkers and apolipoproteins are < 5%. Our oxysterol assay has been validated in accordance with the FDA guidance for bioanalytical methods. The coefficient of variation (CV) was ≤ 8.7% 1. Range and consistency checks will be performed to identify errors in data entry. We will obtain descriptive statistics and assess distributions and pairwise correlations to inform analyses.

# Confidentiality

## **Confidentiality of Study Data**

*Describe the local procedures for maintenance of confidentiality of* ***study data and any records that will be reviewed for data collection****.*

- 1. A. Where and how will all data and records be stored? Include information about: password protection, encryption, physical controls, authorization of access, and separation of identifiers and data, as applicable. Include physical (e.g. paper) **and** electronic files.

Response:

Data and results from the project will be banked for future use in Dr. Ramanathan’s laboratory (Kapoor 357 and Kapoor 355) on secure encrypted computer files. The data will be accessible and available to Dr. Ramanathan’s group, Dr. Browne’s and Dr. Weinstock-Guttman’s groups.

- 1. A. How long will the data be stored?

Response:

The data will be stored indefinitely.

- 1. A. Who will have access to the data?

Response:

Buffalo MS research group comprised of Drs. Murali Ramanathan, Bianca Weinstock-Guttman and Richard Browne at Buffalo. We will share data with Dr. Terry Wahls’ group who are coordinating the patient recruitment. The de-identified results from the project will be made available to qualified investigators interested in MS disease progression and therapy research and to non-profits e.g., National Institutes of Health, National Multiple Sclerosis Society etc. under collaborative arrangements. Specimens will be de-identified.

- 1. A. Who is responsible for receipt or transmission of the data?

Response:

Dr. Ramanathan and his designees

- 1. A. How will the data be transported?

Response:

The data will be transported by electronic means.

## **Confidentiality of Study Specimens**

*Describe the local procedures for maintenance of confidentiality of* ***study specimens****.*

**N/A:** No specimens will be collected or analyzed in this research.
(Skip to Section 19.0)

- 1. B. Where and how will all specimens be stored? Include information about: physical controls, authorization of access, and labeling of specimens, as applicable.

Response:

Specimens from the project will be banked for future use in Dr. Ramanathan’s laboratory (Kapoor 314 and Kapoor 355D) and in Dr. Browne’s laboratory. The laboratories and freezers are secure.

- 1. B. How long will the specimens be stored?

Response:

The specimens remaining after analyses will be stored indefinitely. This is necessary because there is yet no cure for MS.

- 1. B. Who will have access to the specimens?

Response:

The data will be accessible and available to Dr. Ramanathan’s group, Dr. Browne’s and Dr. Weinstock-Guttman’s groups.

- 1. B. Who is responsible for receipt or transmission of the specimens?

Response:

Dr. Ramanathan and Dr. Browne or their research designees will be responsible.

- 1. B. How will the specimens be transported?

Response:

The specimens will be transported manually or by automobile in closed insulated boxes.

# Provisions to Monitor the Data to Ensure the Safety of Subjects

✔ **N/A:** This study is not enrolling subjects, or is limited to records review procedures only. This section does not apply.

**NOTE: Minimal risk studies may be required to monitor subject safety if the research procedures include procedures that present unique risks to subjects that require monitoring. Some examples include: exercising to exertion, or instruments that elicit suicidality or substance abuse behavior. In such cases, N/A is not an acceptable response.**

- 1. Describe the plan to periodically evaluate the data collected regarding both harms and benefits to determine whether subjects remain safe.

Response:

- 1. Describe what data are reviewed, including safety data, untoward events, and efficacy data.

Response:

- 1. Describe any safety endpoints.

Response:

- 1. Describe how the safety information will be collected (e.g., with case report forms, at study visits, by telephone calls with participants).

Response:

- 1. Describe the frequency of safety data collection.

Response:

- 1. Describe who will review the safety data.

Response:

- 1. Describe the frequency or periodicity of review of cumulative safety data.

Response:

- 1. Describe the statistical tests for analyzing the safety data to determine whether harm is occurring.

Response:

- 1. Describe any conditions that trigger an immediate suspension of the research.

Response:

# Withdrawal of Subjects

✔ **N/A:** This study is not enrolling subjects. This section does not apply.

- 1. Describe **anticipated** circumstances under which subjects may be withdrawn from the research without their consent.

Response:

- 1. Describe any procedures for orderly termination.

NOTE: Examples may include return of study drug, exit interview with clinician. Include whether additional follow up is recommended for safety reasons for physical or emotional health.

Response:

- 1. Describe procedures that will be followed when subjects withdraw from the research, including retention of already collected data, and partial withdrawal from procedures with continued data collection, as applicable.

Response:

# Risks to Subjects

- 1. List the reasonably foreseeable risks, discomforts, hazards, or inconveniences to the subjects related to their participation in the research. Consider physical, psychological, social, legal, and economic risks. Include a description of the probability, magnitude, duration, and reversibility of the risks.

NOTE: Breach of confidentiality is always a risk for identifiable subject data.

Response: The research involves no more than minimal risk to the subjects. The forseeable risks include loss of data. However, the data will be de-identified and the direct impact of such an event on subjects’ physical, psychological, social and legal and economic well being estimated to be minimal.

- 1. Describe procedures performed to lessen the probability or magnitude of risks, including procedures being performed to monitor subjects for safety.

Response:

Not applicable. No procedures on subjects.

- 1. If applicable, indicate **which procedures** may have risks to the subjects that are currently unforeseeable.

Response:

Not applicable. No recruitment or procedures on subjects.

- 1. If applicable, indicate which research procedures may have risks to an embryo or fetus should the subject be or become pregnant.

Response:

No applicable. No recruitment.

- 1. If applicable, describe risks to others who are not subjects.

Response:

No risk to others who are not subjects is anticipated.

# Potential Benefits to Subjects

- 1. Describe the potential benefits that individual subjects may experience by taking part in the research. Include the probability, magnitude, and duration of the potential benefits. Indicate if there is no direct benefit.

NOTE: Compensation **cannot** be stated as a benefit.

Response:

No direct benefits to individual subjects.

# Compensation for Research-Related Injury

✔ **N/A:** The research procedures for this study do not present risk of research related injury (e.g. survey studies, records review studies). This section does not apply.

- 1. **If the research procedures carry a risk of research related injury,** describe the available compensation to subjects in the event that such injury should occur.

Response:

- 1. Provide a copy of contract language, if any, relevant to compensation for research related injury.

NOTE: If the contract is not yet approved at the time of this submission, submit the current version here. If the contract is later approved with **different** **language regarding research related injury**, you must modify your response here and submit an amendment to the IRB for review and approval.

Response:

# Economic Burden to Subjects

- 1. Describe any costs that subjects may be responsible for because of participation in the research.

NOTE: Some examples include transportation or parking.

Response:

✔ **N/A:** This study is not enrolling subjects, or is limited to records review procedures only. This section does not apply.

# Compensation for Participation

25.1 Describe the amount and timing of any compensation to subjects, including monetary, course credit, or gift card compensation.

Response:

✔ **N/A:** This study is not enrolling subjects, or is limited to records review procedures only. This section does not apply.

**N/A:** There is no compensation for participation. This section does not apply.

# Consent Process

- 1. Indicate whether you will be obtaining consent.

NOTE: This does not refer to consent documentation, but rather whether you will be obtaining permission from subjects to participate in a research study.
Consent documentation is addressed in Section 27.0.

**Yes** (If yes, Provide responses to each question in this Section)

✔ **No** (If no, Skip to Section 27.0)

- 1. Describe where the consent process will take place. Include steps to maximize subjects’ privacy.

Response:

- 1. Describe how you will ensure that subjects are provided with a sufficient period of time to consider taking part in the research study.

NOTE: It is always a requirement that a prospective subject is given sufficient time to have their questions answered and consider their participation. See “SOP: Informed Consent Process for Research (HRP-090)” Sections 5.5 and 5.6.

Response:

- 1. Describe any process to ensure ongoing consent, defined as a subject’s willingness to continue participation for the duration of the research study.

Response:

- 1. Indicate whether you will be following “SOP: Informed Consent Process for Research (HRP-090).” If not, or if there are any exceptions or additional details to what is covered in the SOP, describe:
     - The role of the individuals listed in the application who are involved in the consent process
     - The time that will be devoted to the consent discussion
     - Steps that will be taken to minimize the possibility of coercion or undue influence
     - Steps that will be taken to ensure the subjects’ understanding

Response:

We have reviewed and will be following “SOP: Informed Consent Process for Research (HRP-090).”

**Non-English Speaking Subjects**

**N/A:** This study will not enroll Non-English speaking subjects.
(Skip to Section 26.8)

- 1. Indicate which language(s) other than English are likely to be spoken/understood by your prospective study population or their legally authorized representatives.

NOTE: The response to this Section should correspond with your response to Section 6.4 of this protocol.

Response:

- 1. If subjects who do not speak English will be enrolled, describe the process to ensure that the oral and written information provided to those subjects will be in that language. Indicate the language that will be used by those obtaining consent.

NOTE: Guidance is provided on “SOP: Informed Consent Process for Research (HRP-090).”

Response:

**Cognitively Impaired Adults**

**N/A**: This study will not enroll cognitively impaired adults.
*(Skip to Section 26.9)*

- 1. Describe the process to determine whether an individual is capable of consent.

Response:

**Adults Unable to Consent**

**N/A**: This study will not enroll adults unable to consent.
(*Skip to Section 26.13)*

When a person is not capable of consent due to cognitive impairment, a legally authorized representative should be used to provide consent (Sections 26.9 and 26.10) **and, where possible, assent of the individual should also be solicited** (Sections 26.11 and 26.12).

- 1. Describe how you will identify a Legally Authorized Representative (LAR). Indicate that you have reviewed the “SOP: Legally Authorized Representatives, Children, and Guardians (HRP-013)” for research in New York State.

NOTE: Examples of acceptable response includes: verifying the electronic medical record to determine if an LAR is recorded.

Response:

We have reviewed and will be following “SOP: Legally Authorized Representatives, Children, and Guardians (HRP-013).”

- 1. **For research conducted outside of New York State**, provide information that describes which individuals are authorized under applicable law to consent on behalf of a prospective subject to their participation in the research. One method of obtaining this information is to have a legal counsel or authority review your protocol along with the definition of “legally authorized representative” in “SOP: Legally Authorized Representatives, Children, and Guardians (HRP-013).”

Response:

- 1. Describe the process for **assent of the** **adults**:
     - Indicate whether assent will be obtained from all, some, or none of the subjects. If some, indicate which adults will be required to assent and which will not.

Response:

- - - If assent will not be obtained from some or all subjects, provide an explanation of why not.

Response:

- 1. Describe whether **assent of the** **adult** subjects will be documented and the process to document assent.

NOTE: The IRB allows the person obtaining assent to document assent on the consent document using the “Template Consent Document (HRP-502)” Signature Block for Assent of Adults who are Legally Unable to Consent.

Response:

**Subjects who are not yet Adults (Infants, Children, and Teenagers)**

**N/A**: This study will not enroll subjects who are not yet adults.
*(Skip to Section 27.0)*

- 1. Describe the criteria that will be used to determine **whether a prospective subject has not attained the legal age for consent to treatments or procedures involved in the research** under the applicable law of the jurisdiction in which the research will be conducted **(e.g., individuals under the age of 18 years)**. For research conducted in NYS, review “SOP: Legally Authorized Representatives, Children, and Guardians (HRP-013)” to be aware of which individuals in the state meet the definition of “children.”

NOTE: Examples of acceptable responses include: verification via electronic medical record, driver’s license or state-issued ID, screening questionnaire.

Response:

- 1. **For research conducted outside of New York State**, provide information that describes which persons have not attained the legal age for consent to treatments or procedures involved the research, under the applicable law of the jurisdiction in which research will be conducted. One method of obtaining this information is to have a legal counsel or authority review your protocol along the definition of “children” in “SOP: Legally Authorized Representatives, Children, and Guardians (HRP-013).”

Response:

- 1. Describe whether parental permission will be obtained from:

Response:

One parent even if the other parent is alive, known, competent, reasonably available, and shares legal responsibility for the care and custody of the child.

Both parents unless one parent is deceased, unknown, incompetent, or not reasonably available, or when only one parent has legal responsibility for the care and custody of the child.

Parent permission will not be obtained. A waiver of parent permission is being requested.

NOTE: The requirement for parent permission is a protocol-specific determination made by the IRB based on the risk level of the research. For guidance, review the “CHECKLIST: Children (HRP-416).”

- 1. Describe whether permission will be obtained from individuals **other than parents**, and if so, who will be allowed to provide permission. Describe your procedure for determining an individual’s authority to consent to the child’s general medical care.

Response:

- 1. Indicate whether assent will be obtained from all, some, or none of the **children**. If assent will be obtained from some children, indicate which children will be required to assent.

Response:

- 1. When assent of children is obtained, describe how it will be documented.

Response:

# Waiver or Alteration of Consent Process

**Consent will not be obtained, required information will not be disclosed, or the research involves deception.**

✔ A waiver or alteration of consent is not being requested.

- 1. If the research involves a waiver or alteration of the consent process, please review the “CHECKLIST: Waiver or Alteration of Consent Process (HRP-410)” to ensure that you have provided sufficient information for the IRB to make the determination that a waiver or alteration can be granted.

NOTE: For records review studies, the first set of criteria on the “CHECKLIST: Waiver or Alteration of Consent Process (HRP-410)” applies.

Response:

- 1. If the research involves a waiver of the consent process for planned emergency research, please review the “CHECKLIST: Waiver of Consent for Emergency Research (HRP-419)” to ensure you have provided sufficient information for the IRB to make these determinations. Provide any additional information necessary here:

Response:

# Process to Document Consent

**N/A:** A Waiver of Consent is being requested.
(Skip to Section 29.0)

- 1. Indicate whether you will be following “SOP: Written Documentation of Consent (HRP-091).” If not or if there are any exceptions, describe whether and how consent of the subject will be obtained including whether or not it will be documented in writing.

NOTE: If your research presents no more than minimal risk of harm to subjects and involves no procedures for which written documentation of consent is normally required outside of the research context, the IRB will generally waive the requirement to obtain written documentation of consent. This is sometimes referred to as ‘verbal consent.’ Review “CHECKLIST: Waiver of Written Documentation of Consent (HRP-411)” to ensure that you have provided sufficient information.


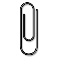
If you will document consent in writing, attach a consent document with your submission. You may use “TEMPLATE CONSENT DOCUMENT (HRP-502)”. If you will obtain consent, but not document consent in writing, attach the script of the information to be provided orally or in writing (i.e. consent script or Information Sheet).

Response:

We will be following “SOP: Written Documentation of Consent”
(HRP-091).

# Multi-Site Research (Multisite/Multicenter Only)

✔ **N/A:** This study is not an investigator-initiated multi-site study. This section does not apply.

- 1. If this is a multi-site study **where you are the lead investigator**, describe the processes to ensure communication among sites, such as:
     - All sites have the most current version of the IRB documents, including the protocol, consent document, and HIPAA authorization.
     - All required approvals have been obtained at each site (including approval by the site’s IRB of record).
     - All modifications have been communicated to sites, and approved (including approval by the site’s IRB of record) before the modification is implemented.
     - All engaged participating sites will safeguard data as required by local information security policies.
     - All local site investigators conduct the study appropriately.
     - All non-compliance with the study protocol or applicable requirements will be reported in accordance with local policy.

Response:

- 1. Describe the method for communicating to engaged participating sites:
     - Problems
     - Interim results
     - Study closure

Response:

- 1. Indicate the total number of subjects that will be enrolled or records that will be reviewed across all sites.

Response:

- 1. If this is a multicenter study for which UB will serve as the IRB of record, and subjects will be recruited by methods not under the control of the local site (e.g., call centers, national advertisements) describe those methods.

Response:

# Banking Data or Specimens for Future Use

**N/A:** This study is not banking data or specimens for future use or research outside the scope of the present protocol. This section does not apply.

- 1. If data or specimens will be banked (stored) for **future use, that is, use or research outside of the scope of the present protocol**, describe where the data/specimens will be stored, how long they will be stored, how the data/specimens will be accessed, and who will have access to the data/specimens.

NOTE: Your response here must be consistent with your response at the “What happens if I say yes, I want to be in this research?” Section of the Template Consent Document (HRP-502).

Response:

Data and specimens from the project will be banked for future use in Dr. Ramanathan’s laboratory (Kapoor 357, Kapoor 355, Kapoor 314) on secure encrypted computer files or freezers The data will be accessible and available to Dr. Ramanathan’s group, Dr. Browne’s, and Dr. Weinstock-Guttman’s groups. The data and specimens will be stored indefinitely.

- 1. List the data to be stored or associated with each specimen.

Response:

The data on cholesterol and lipid profiles, cholesterol biomarkers, cholesteryl esters profile, paraoxonase activities, oxysterol profiles will be stored alongside the data collected from Dr. Wahls’ study for each subject’s sample.

- 1. Describe the procedures to release banked data or specimens for future uses, including: the process to request a release, approvals required for release, who can obtain data or specimens, and the data to be provided with specimens.

Response:

The data will be freely shared between the collaborating groups (The Buffalo MS research group comprised of Drs. Murali Ramanathan, Bianca Weinstock-Guttman, and Richard Browne at Buffalo. The data will be shared with Dr. Wahls’ group. The de-identified results from the project will be made available to qualified investigators interested in MS disease progression and therapy research and to non-profits such as the National Multiple Sclerosis Society under collaborative arrangements. Specimens will be de-identified.

# Drugs or Devices

✔ **N/A:** This study does not involve drugs or devices. This section does not apply.

- 1. If the research involves drugs or devices, list and describe all drugs and devices used in the research, the purpose of their use, and their regulatory approval status.

Response:

- 1. Describe your plans to store, handle, and administer those drugs or devices so that they will be used only on subjects and be used only by authorized investigators.

Response:

**If the drug is investigational (has an IND) or the device has an IDE or a claim of abbreviated IDE (non-significant risk device), include the following information:**

- 1. Identify the holder of the IND/IDE/Abbreviated IDE.

Response:

- 1. Explain procedures followed to comply with FDA sponsor requirements for the following:

|  | ***Applicable to:*** | | |
| --- | --- | --- | --- |
| ***FDA Regulation*** | ***IND Studies*** | ***IDE studies*** | ***Abbreviated IDE studies*** |
| ***21 CFR 11*** | ***X*** | ***X*** |  |
| ***21 CFR 54*** | ***X*** | ***X*** |  |
| ***21 CFR 210*** | ***X*** |  |  |
| ***21 CFR 211*** | ***X*** |  |  |
| ***21 CFR 312*** | ***X*** |  |  |
| ***21 CFR 812*** |  | ***X*** | ***X*** |
| ***21 CFR 820*** |  | ***X*** |  |

Response:

# Humanitarian Use Devices

✔ **N/A:** This study does not involve humanitarian use devices. This does not apply.

- 1. For Humanitarian Use Device (HUD) uses provide a description of the device, a summary of how you propose to use the device, including a description of any screening procedures, the HUD procedure, and any patient follow-up visits, tests or procedures.

Response:

- 1. For HUD uses provide a description of how the patient will be informed of the potential risks and benefits of the HUD and any procedures associated with its use.

Response:
